# Association of Immune-Mediated Inflammatory Diseases and Fracture Risk in Patients with Type 2 Diabetes: A Nationwide Population-Based Study

**DOI:** 10.3390/jcm14030795

**Published:** 2025-01-25

**Authors:** Yuna Kim, Oh Chan Kwon, Ryul Kim, Jong Hoon Kim, Min-Jae Kim, Min-Chan Park, Jie-Hyun Kim, Young Hoon Youn, Hyojin Park, Kyungdo Han, Jaeyoung Chun

**Affiliations:** 1 Division of Gastroenterology, Department of Internal Medicine, Gangnam Severance Hospital, Yonsei University College of Medicine, Seoul 06229, Republic of Korea; sadts@yuhs.ac (Y.K.); kmj0630@yuhs.ac (M.-J.K.); otilia94@yuhs.ac (J.-H.K.); dryoun@yuhs.ac (Y.H.Y.); hjpark21@yuhs.ac (H.P.); 2 Division of Rheumatology, Department of Internal Medicine, Gangnam Severance Hospital, Yonsei University College of Medicine, Seoul 06229, Republic of Korea; ockwon@yuhs.ac (O.C.K.); mcpark@yuhs.ac (M.-C.P.); 3 Department of Neurology, Inha University Hospital, Incheon 22332, Republic of Korea; arkrk86@inha.ac.kr; 4 Department of Dermatology and Cutaneous Biology Research Institute, Gangnam Severance Hospital, Yonsei University College of Medicine, Seoul 06229, Republic of Korea; jhkim074@yuhs.ac; 5 Department of Statistics and Actuarial Science, Soongsil University, Seoul 06978, Republic of Korea

**Keywords:** bone fractures, diabetes mellitus, healthcare administrative claims, immune-mediated inflammatory disease

## Abstract

**Background**: Immune-mediated inflammatory diseases (IMIDs) are associated with chronic inflammation that may increase fracture risk; however, their impact within specific populations, such as those with type 2 diabetes mellitus (T2DM), remains unclear. This study aimed to evaluate the association between IMIDs and fracture risk, leveraging a large, high-quality population-based cohort of patients with T2DM. **Methods**: A total of 2,120,900 patients with T2DM without a previous history of fractures were enrolled from the nationwide health check-up database provided by the Korean National Health Insurance Service. The outcomes were overall, osteoporotic, non-osteoporotic, vertebral and hip fractures. Multivariable Cox proportional hazard regression analysis was performed to estimate the adjusted hazard ratios (aHRs) with 95% confidence intervals (CIs) according to the presence of IMIDs. **Results**: The presence of ulcerative colitis (aHR: 1.31), rheumatoid arthritis (aHR: 1.19), ankylosing spondylitis (aHR: 1.32), and psoriasis (aHR: 1.14) were significantly associated with the risk of overall fractures. Compared with controls, patients with a single IMID (aHR: 1.18) and at least two IMIDs (aHR: 1.29) had a significantly increased risk of overall fractures, showing a dose–response relationship. Similar results were observed for osteoporotic, vertebral, and hip fractures. **Conclusions**: The presence of IMIDs in patients with T2DM was associated with an increased risk of fractures, particularly osteoporotic, vertebral, and hip fractures. This study highlights the significant impact of IMIDs on fracture risk within a diabetic population, emphasizing the need for careful monitoring and tailored management strategies.

## 1. Introduction

Increased bone fragility is a common and severe complication of type 2 diabetes mellitus (T2DM), particularly among the elderly who have multiple risk factors for falls and fractures [1]. Although the pathophysiology underlying the effect of T2DM on fractures remains unclear, evidence suggests that diabetes is associated with a higher fracture risk [2,3,4,5,6]. The potential mechanistic pathways underlying the link between T2DM and fractures involve several factors. Chronic hyperglycemia, oxidative stress, and reduced insulin signaling impair bone strength and remodeling, contributing to this increased risk [7,8].

Immune-mediated inflammatory diseases (IMIDs) are a heterogeneous category of diseases that cause chronic inflammation and organ damage and include inflammatory bowel disease (Crohn’s disease [CD] and ulcerative colitis [UC]), rheumatoid arthritis (RA), ankylosing spondylitis (AS), and psoriasis (PsO) [9]. Previous studies have reported an association between each IMID and the fracture risk in the general population [10,11,12,13,14,15,16,17]. The chronic inflammation characteristic of IMIDs is thought to influence bone health by disrupting bone remodeling, promoting osteoclastogenesis, and reducing osteoblast function. Pro-inflammatory cytokines such as tumor necrosis factor-alpha (TNF-α) and interleukin-6 (IL-6) may further impair bone quality, and when combined with T2DM-related metabolic abnormalities, may amplify skeletal fragility in affected individuals [18].

Although several studies have investigated the impact of IMIDs or T2DM on fracture risk individually, the combined effect of these conditions has not been thoroughly evaluated [3,13,14,17]. It is essential to determine the effect of IMIDs on the fracture risk in patients with T2DM, as this may lead to fracture risk stratification in these patients. This study utilizes a nationwide, high-quality dataset of T2DM patients to explore how IMIDs contribute to fracture risk, providing novel insights into this significant research gap.

## 2. Methods

### 2.1. Data Source

This nationwide population-based study used data from the claims database of the Korean National Health Insurance Service (NHIS), which includes demographic characteristics, socioeconomic status, usage of medical services, and comorbidities according to the International Classification of Diseases, Tenth Revision (ICD-10), and rare intractable disease (RID) registration information [19]. In the Korean RID system, diagnoses are based on the NHIS standardized diagnostic criteria, which are thoroughly reviewed by the healthcare institution and NHIS before registration. All participants registered in the NHIS are advised to undergo national health checkups biannually, which include measurements such as anthropometric data, blood pressure, and laboratory data such as serum fasting glucose, cholesterol, and creatinine levels. Data on medical history and lifestyle factors such as smoking, alcohol consumption, and physical activity were collected using standardized self-reporting questionnaires. The Institutional Review Board (IRB) of Gangnam Severance Hospital approved this study (IRB No:3-2020-0269). The requirement for informed consent was waived due to the retrospective study design.

### 2.2. Study Cohort

**Figure 1:**
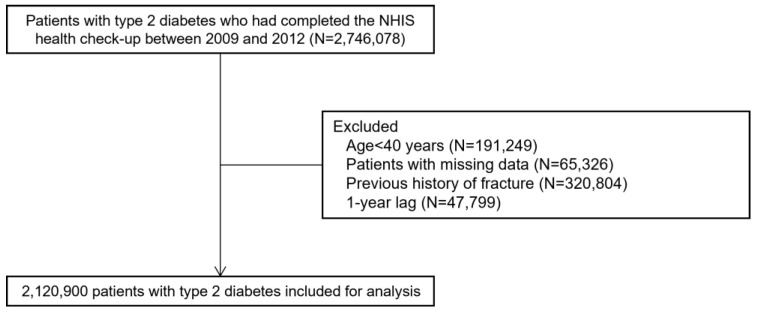
Selection of the study population. NHIS—National Health Insurance Service.

From the general health checkup program, we selected 2,746,078 subjects with T2DM who underwent a health examination between 1 January 2009 and 31 December 2012 (Figure 1). If a subject underwent two or more examinations between 2009 and 2012, data from the first examination were used for the analysis. The following criteria were used to determine subjects with T2DM: (1) the presence of ICD-10 codes E11–14 and claims for at least one oral antidiabetic medication or insulin at baseline or (2) a fasting glucose level ≥ 126 mg/dL (obtained from the health examination database).

The following were exclusion criteria: (i) age <40 years (n = 191,249); (ii) missing data (n = 65,326); (iii) previous fracture (n = 320,804); and (iv) fractures within 1 year from the date of the first health checkup between 1 January 2009 and 31 December 2012 (n = 47,799). The phrase ‘1 year lag’ refers to the exclusion of fractures occurring within the first year after the baseline health checkup to minimize the effect of pre-existing conditions or fractures unrelated to the diagnosis of T2DM or IMIDs.

Ultimately, 2,120,900 patients with T2DM were included in the analysis. Patients were followed up from baseline (i.e., date of the first health checkup between 1 January 2009 and 31 December 2012) to the date of incident fractures or 31 December 2019, whichever came first.

### 2.3. Outcomes

The study outcomes included overall osteoporotic, non-osteoporotic, vertebral, and hip fractures. All fractures were defined as hospital visits resulting in ICD-10 codes for fractures. Osteoporotic fractures were defined as fractures in four specific parts: spine, proximal humerus, femur, and distal radius, with a diagnosis of osteoporosis before or within 90 days of the fracture [20]. Non-osteoporotic fractures were defined as overall fractures excluding osteoporotic fractures. Definitions of covariates and outcomes are described in the Methods section of the Supplement Materials.

### 2.4. Statistical Analysis

Continuous variables are reported as mean ± standard deviation, and categorical variables are expressed as numbers (%). The independent Student’s t-test was used to compare continuous variables, and the χ^2^ test was used to compare categorical variables. The incidence rate of the outcomes was presented as the number of events per 1000 person-years. Kaplan–Meier curve analysis was used to visualize the cumulative incidence of outcomes based on the presence of IMIDs, and the log-rank test was used to compare the results. The study utilized Cox proportional hazard models to estimate the hazard ratio (HR) and 95% confidence interval (CI) for the outcomes based on (i) the presence of IMIDs (CD, UC, RA, AS, and PsO) and (ii) the number of IMIDs present (0, 1, and ≥2). Five models were constructed for analysis. Model 1 was a univariate model without adjustments, and model 2 was adjusted for age and sex. The subsequent models (models 3, 4, and 5) were adjusted for other potential confounders. The final model (model 5) was adjusted for age, sex, smoking status, alcohol consumption, regular physical activity, hypertension, dyslipidemia, body mass index, depression, insulin use, number of oral hypoglycemic agents, and T2DM duration. Subgroup analyses were conducted by stratifying the patients based on several covariates and interaction tests were performed to determine whether the associations differed by other covariates. All *p*-values were two-sided, and a *p*-value < 0.05 was considered significant. All statistical analyses were performed using SAS version 9.4 (SAS Institute, Cary, NC, USA).

## 3. Results

### 3.1. Baseline Characteristics

The baseline characteristics of the 2,120,900 patients with T2DM and a comparison of their characteristics according to the presence of each IMID are reported in Table 1. The mean age of the study population was 58.5 ± 10.5 years, and 1,281,181 (60.4%) patients were male. Of the 2,120,900 patients with T2DM, CD, UC, RA, AS, and PsO were present in 138 (0.01%), 870 (0.04%), 65,696 (3.1%), 657 (0.03%), and 19,693 (0.93%) patients, respectively. Patients with AS were significantly younger than their respective controls, whereas those with UC, RA, or PsO were significantly older. Significantly more men had UC, AS, and PsO, whereas significantly more women had RA than in their respective controls. The proportion of current smokers was significantly lower in patients with CD, UC, and RA but significantly higher in those with AS and PsO compared with their respective controls. Compared to the control group, the proportion of heavy alcohol drinkers was significantly lower in patients with CD, UC, and RA. The proportion of individuals exercising regularly was significantly lower in the patients with RA and AS than in their respective controls. The presence of baseline comorbidities varied among patients with each IMID. The prevalence of osteoporosis was significantly higher in all IMID groups than in controls.

### 3.2. Incidence and Risk of Overall Fractures According to the Presence of IMIDs

**Figure 2:**
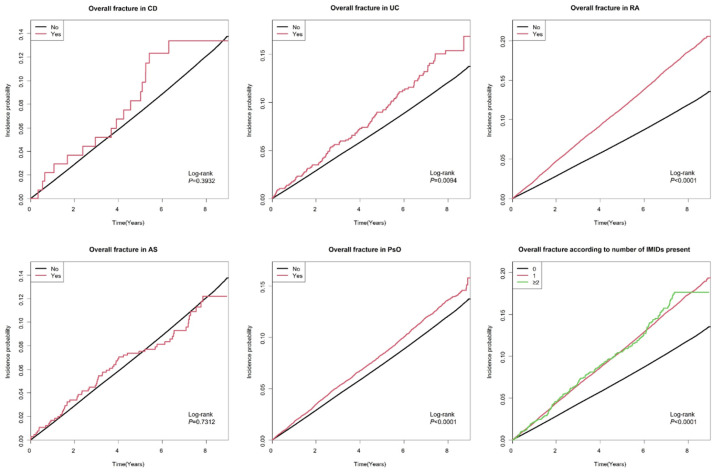
Cumulative incidence of overall fractures according to the presence of each IMID and the number of comorbid IMIDs. CD, Crohn’s disease; UC, ulcerative colitis; RA, rheumatoid arthritis; AS, ankylosing spondylitis; PsO, psoriasis; IMIDs, immune-mediated inflammatory disease.

The incidence rates of overall fractures in patients with T2DM with CD, UC, RA, AS, and PsO were 19.10, 20.22, 25.24, 15.16, and 17.99 per 1000 person-years, respectively (Table 2 and Figure 2). The cumulative incidence of all fractures according to the presence of each IMID and the number of comorbid IMIDs is shown in Figures S1–S4 in Supplement. After adjusting for multiple covariates in the final model (Model 5), the presence of comorbid UC (adjusted HR [aHR]: 1.31 [95%CI: 1.09–1.57]), RA (aHR: 1.19 [95%CI: 1.17–1.22]), AS (aHR: 1.32 [95%CI: 1.03–1.68]), and PsO (aHR: 1.14 [95%CI: 1.10–1.19]) was significantly associated with an increased overall fracture risk in patients with T2DM. The presence of CD showed a trend toward a higher overall fracture risk in patients with T2DM, although the difference was not significant (aHR: 1.23 [95%CI: 0.76–1.97]). Those with one IMID (aHR: 1.18 [95%CI: 1.16–1.21]) had a significantly higher overall fracture risk than those without any IMIDs, and those with at least two IMIDs (aHR: 1.29 [95%CI: 1.11–1.50]) had a significantly higher overall fracture risk than those without any IMIDs, with a larger effect size.

### 3.3. Incidence and Risk of Osteoporotic and Non-Osteoporotic Fractures According to the Presence of IMIDs

The incidence rates of osteoporotic fractures in patients with T2DM with CD, UC, RA, AS, and PsO were 12.36, 10.11, 17.86, 8.53, and 9.15 per 1000 person-years, respectively (Table 3). In the final model (Model 5), after adjusting for multiple covariates, the presence of UC (aHR: 1.34 [95%CI: 1.04–1.74]), RA (aHR: 1.38 [95%CI: 1.35–1.41]), AS (aHR: 1.95 [95%CI: 1.41–2.70]), and PsO (aHR: 1.22 [95%CI: 1.15–1.29]) was significantly associated with an increased osteoporotic fracture risk in patients with T2DM, respectively. The presence of CD showed a trend toward a higher osteoporotic fracture risk in patients with T2DM, although this difference was not significant (aHR: 1.48 [95%CI: 0.82–2.67]). According to the number of IMIDs, T2DM patients with one IMID (aHR: 1.36 [95%CI: 1.33–1.39]) had a significantly higher osteoporotic fracture risk than those without any IMIDs, and those with at least two IMIDs (aHR: 1.53 [95%CI: 1.26–1.84]) also had a significantly higher osteoporotic fracture risk than those without any IMIDs, with a larger effect size.

The incidence rates of non-osteoporotic fractures in patients with T2DM who had CD, UC, RA, AS, and PsO were 6.74, 10.11, 7.38, 6.63, and 8.84 per 1000 person-years, respectively (Table 3). In the final model (Model 5), after adjusting for multiple covariates, the presence of PsO (aHR: 1.11 [95%CI: 1.05–1.18]) was significantly associated with an increased risk of non-osteoporotic fractures, whereas comorbid RA (aHR: 0.92 [95%CI: 0.88–0.95]) was significantly associated with a decreased risk of non-osteoporotic fractures in patients with T2DM.

### 3.4. Incidence and Risk of Vertebral and Hip Fractures According to the Presence of IMIDs

The incidence rates of vertebral fractures in patients with T2DM with CD, UC, RA, AS, and PsO were 3.22, 6.08, 8.55, 5.94, and 5.72 per 1000 person-years, respectively (Table 4). In the final model (Model 5), adjusted for multiple covariates, the presence of UC (aHR: 1.39 [95%CI: 1.00–1.93]), RA (aHR: 1.30 [95%CI: 1.26–1.34]), AS (aHR: 2.11 [95%CI: 1.44–3.11]), and PsO (aHR: 1.29 [95%CI: 1.20–1.38]), but not CD (aHR: 0.70 [95%CI: 0.23–2.16]), was significantly associated with an increased vertebral fracture risk in patients with T2DM.

The incidence rates of hip fractures in T2DM patients with CD, UC, RA, AS, and PsO were 3.19, 2.01, 3.16, 2.02, and 2.16 per 1000 person-years, respectively (Table 4). In the final model (Model 5), adjusted for multiple covariates, the presence of RA (aHR: 1.19 [95%CI: 1.13–1.26]), AS (aHR: 2.06 [95%CI: 1.07–3.96]), and PsO (aHR: 1.16 [95%CI: 1.03–1.31]) was significantly associated with an increased hip fracture risk.

T2DM patients with one IMID had a significantly higher vertebral fracture risk (aHR: 1.30 [95%CI: 1.26–1.34]) and hip fracture risk (aHR: 1.19 [95%CI: 1.13–1.25]) than those without any IMIDs. Also, those with at least two IMIDs had a significantly higher vertebral fracture risk (aHR: 1.51 [95%CI: 1.18–1.94]) and hip fracture risk (aHR: 1.31 [95%CI: 0.86–1.98]) than those without any IMIDs, with a larger effect size.

### 3.5. Subgroup Analyses

Based on the number of comorbid IMIDs, the fracture risk was demonstrated in all subgroups, including age, sex, smoking behavior, alcohol consumption, regular physical activity, and disease duration of T2DM. Among T2DM patients who exercised regularly, the impact of comorbid IMIDs on the overall fracture risk showed a tendency to subside (Table S1 in Supplement). The preventive effect of regular physical activity regarding overall fractures was significant only in T2DM patients with comorbid PsO, and a significant interaction was observed for osteoporotic fracture risk among patients with T2DM and PsO. The effect of PsO on the development of osteoporotic and vertebral fractures was significantly more pronounced in male patients with T2DM. The impact of PsO on the risk of non-osteoporotic fractures and hip fractures was similar across different subgroups (Tables S2–S6 in Supplement).

The impact of RA on the overall development of fractures was significantly more pronounced in males, former or current smokers, and patients with T2DM for <5 years. In terms of the risk of osteoporotic fractures, the impact of comorbid RA was significantly more pronounced in T2DM patients aged <65 years, males, former or current smokers, alcohol drinkers, and those with T2DM duration <5 years. The impact of comorbid RA on the development of non-osteoporotic fractures was significantly more prominent in T2DM patients aged <65 years, males, former or current smokers, and alcohol drinkers. The impact of comorbid RA on the development of vertebral fractures was significantly more pronounced in T2DM patients aged <65 years, males, former or current smokers, and those with a T2DM duration <5 years. The impact of RA on the development of hip fractures was significantly more pronounced in patients with a T2DM duration <5 years.

There were no significant differences in the impact of CD, UC, and AS on the risk of fracture among the subgroups.

## 4. Discussion

This nationwide population-based study of two million patients with T2DM found that the presence of IMIDs was associated with a significantly higher overall fracture risk in patients with T2DM. In particular, UC, RA, AS, and PsO were significantly associated with an increased overall fracture risk in patients with T2DM. The presence of comorbid CD was also associated with a 1.2-fold increase in the overall fracture risk in patients with T2DM. Similar associations between each IMID and osteoporotic, but not non-osteoporotic, fractures were observed in patients with T2DM. Moreover, increasing trends in the risk of overall, osteoporotic, vertebral, and hip fractures, but not non-osteoporotic fractures, were observed according to the number of comorbid IMIDs in patients with T2DM. These findings provide evidence of the clinical impact of IMIDs on bone metabolism in T2DM patients, emphasizing their compounding effects on fracture risk.

To our knowledge, this is the first population-based study to assess the association between fractures and IMIDs in patients with T2DM. Bone formation is impaired in T2DM due to chronic inflammation, advanced glycation end products, and oxidative stress. These factors, combined with IMID-related systemic inflammation, further disrupt bone healing processes [21,22]. However, diabetes itself represents a pro-inflammatory condition, often characterized by altered immune responses involving both innate and acquired immunity. This pro-inflammatory state may predispose patients with T2DM to a higher prevalence of IMIDs, which in turn could further exacerbate fracture risk through chronic inflammation and its effects on bone health [23].

In this study, comorbid RA was associated with a 19% increased risk of overall fracture in patients with T2DM. Although the effect size was smaller than that in the general population reported in a previous meta-analysis (1.5-fold increased risk) [14], given that patients with T2DM already have a higher fracture risk [1], the increased risk observed in our study was considerable. Based on the fracture site, RA is associated with an increased risk of both vertebral and hip fractures. RA is known to impair the trabecular and cortical bones [24], which could explain the increased fracture risk at both sites. Additionally, in our analysis, the protective effect of rheumatoid arthritis (RA) on non-osteoporotic fractures was observed. One previous study identified fracture risk factors in the non-osteoporotic elderly population, which included low BMD, advancing age, falls during the last 12 months, and prior fracture history. In our study, all patients with a prior fracture history were excluded, and there was no information on BMD and fall events, which are known risk factors [25]. This observed protective effect of RA on non-osteoporotic fractures is likely due to unmeasured confounders, such as fall history or undiagnosed bone conditions, rather than a true biological phenomenon. Subgroup analysis indicated that men with both T2DM and RA appeared to have a higher risk of overall, osteoporotic, and non-osteoporotic fractures compared to women. While the reasons for this observation are not entirely clear, it is possible that the combined effects of T2DM-related changes in bone quality, RA-associated systemic inflammation, and lifestyle factors such as higher smoking and alcohol consumption rates among men may contribute to this trend. However, given the unexpected nature of this finding and the lack of definitive evidence, further research is needed to better understand the influence of sex differences on fracture risk in patients with both T2DM and RA [14].

AS was associated with a 32% increased risk of overall fractures in patients with T2DM. In this study, AS was significantly associated with an increased risk of osteoporotic, vertebral, and hip fractures but not non-osteoporotic fractures. This is consistent with previous studies reporting an increased risk of vertebral and hip fractures in patients with AS [26,27]. Notably, the effect size of AS regarding osteoporotic, vertebral, and hip fractures was the largest among all IMIDs, with a twofold increased risk of each outcome. AS is a chronic inflammatory disease characterized by bony ankylosis of the axial skeleton, leading to stiffness and reduced mobility, which could explain the striking impact of AS on fractures in patients with T2DM. However, regular follow-ups with spine and hip X-rays in AS patients could lead to detection bias, as compression fractures often occur without symptoms. The higher HR for vertebral fractures in AS patients in our study may indicate increased detection due to more frequent monitoring, rather than a higher actual incidence of fractures. Nonetheless, based on our results, AS should be considered the IMID with the strongest impact on fractures in patients with T2DM, thus requiring monitoring.

PsO was associated with a 14% increased risk of overall fractures in patients with T2DM. Moreover, PsO is significantly associated with an increased risk of osteoporotic, non-osteoporotic, vertebral, and hip fractures. This is consistent with a previous meta-analysis, which showed an increased fracture risk in patients with PsO [28]. Interestingly, the impact of PsO on the development of overall and osteoporotic fractures was not significant in T2DM patients who exercised regularly, suggesting the positive effect of regular physical activity on the prevention of fractures related to comorbid skin IMIDs. However, we recognize that our analysis does not fully capture the nuances of disease severity, medication use, or the direct effects of physical activity itself, making it difficult to draw definitive conclusions about the reliability of these results. Further prospective studies are warranted to evaluate whether regular physical activity reduces the risk of overall and osteoporotic fractures associated with IMIDs in T2DM patients.

UC was associated with a 31% increased risk of overall fractures in patients with T2DM. The higher risk of overall fractures was attributable to osteoporotic fractures but not to non-osteoporotic fractures. This finding suggests that special attention is needed, particularly in patients with T2DM who have concomitant UC and osteoporosis. Concerning the anatomical location of fractures, UC was significantly associated with an increased risk of vertebral fractures but not hip fractures, which might be associated with trabecular bone loss in patients with UC, as reported previously [29]. CD showed a trend towards a higher overall fracture risk in patients with T2DM, but this was not significant owing to the small number of patients with CD. Previous studies reported a significantly higher fracture risk in patients with CD than in the general population [10]. Possibly, the effect of CD on the relative fracture risk might be attenuated in the specific population of T2DM patients at risk of fractures [1]. Further large-scale studies are required to validate whether CD carries an additional fracture risk in T2DM.

This study had some limitations. First, the effect of medications on IMIDs was not considered. It is unclear whether the higher fracture risk was due to IMIDs or the medications for IMIDs. However, separate studies have shown that in T2DM, steroid use increases fracture risk, and independently, for IMIDs, especially in IBD, steroid use also increase fracture risk [30]. Therefore, it is plausible that the combination of T2DM, IMIDs, and steroid use could further amplify fracture risk, and this requires future investigation. Anti-osteoporotic medication use, such as bisphosphonates, vitamin D, or calcium supplementation, was also not accounted for, despite their known preventive effects on fractures [31,32]. Second, the causal relationship between IMIDs and fractures may be unclear because the presence of IMIDs is associated with more prevalent manifestations of bone diseases that require diagnostic examination. Third, osteoporosis was defined using ICD-10 codes (M80–M82), which may underestimate the actual prevalence due to the frequent use of osteopenia codes (M85) in Korea.

The lack of BMD data further compounds this issue, as BMD is a critical determinant of fracture risk. Without these data, our ability to distinguish between low bone mass and normal bone density fractures is limited, potentially leading to misclassification of fracture types and confounding the results. Fourth, while we included individuals aged 40 and above to capture a broader at-risk population, defining osteoporosis in younger adults—particularly men under 50 and premenopausal women—remains challenging and may have influenced the study’s outcomes. Additionally, the lack of information on fall events, a key fracture contributor, and the exclusion of patients with prior fractures may limit generalizability as they represent a high-risk group. Furthermore, we did not stratify fracture risk by IMID severity or include measures like HbA1c, which may directly influence fracture risk. Future research should explore the impact of specific treatments, such as biologics, DMARDs, and anti-osteoporotic therapies, on fracture risk in patients with T2DM and IMIDs.

In conclusion, using a large population-based cohort, the presence of IMIDs in T2DM patients was associated with a higher fracture risk. These findings highlight the importance of considering IMIDs as critical factors in fracture risk assessments, particularly in populations with T2DM. Tailored management strategies, such as enhanced bone density screening, targeted preventive measures, and lifestyle interventions, are critical to mitigate fracture risks. These strategies should be integrated into routine care to address this high-risk population effectively.

## Figures and Tables

**Table 1:** Baseline characteristics of the patients with type 2 diabetes according to the presence of immune-mediated inflammatory diseases.

	Total Population	No CD	CD	*p*	No UC	UC	*p*	No RA	RA	*p*	No AS	AS	*p*	No PsO	PsO	*p*
Number of patients	2,120,900	2,120,762	138		2,120,030	870		2,055,204	65,696		2,120,243	657		2,101,207	19,693	
Age, years, mean ± SD	58.47 ± 10.53	58.47 ± 10.53	57.16 ± 10.93	0.144	58.47 ± 10.53	59.23 ± 10.22	0.034	58.36 ± 10.54	61.89 ±9.8	<0.001	58.47 ± 10.53	54.53 ± 9.98	<0.001	58.46 ± 10.53	59.68 ± 10.32	<0.001
Age, years, n (%)				0.535			0.040			<0.001			<0.001			<0.001
40–65	1,495,743 (70.52)	1,495,649 (70.52)	94 (68.12)		1,495,157 (70.53)	586 (67.36)		1,456,938 (70.89)	38,805 (59.07)		1,495,205 (70.52)	538 (81.89)		1,482,567 (70.56)	13,176 (66.91)	
65≤	625,157 (29.48)	625,113 (29.48)	44 (31.88)		624,873 (29.47)	284 (32.64)		598,266 (29.11)	26,891 (40.93)		625,038 (29.48)	119 (18.11)		618,640 (29.44)	6517 (33.09)	
Male sex, n (%)	1,281,181 (60.41)	1,281,091 (60.41)	90 (65.22)	0.248	1,280,574 (60.40)	607 (69.77)	<0.001	1,257,075 (61.17)	24,106 (36.69)	<0.001	1,280,645 (60.40)	536 (81.58)	<0.001	1,267,810 (60.34)	13371 (67.90)	<0.001
BMI, kg/m^2^, Mean ± SD	25.07 ±3.29	25.07 ± 3.29	23.78 ± 3.53	<0.001	25.07 ± 3.29	24.4 ± 2.99	<0.001	25.07 ± 3.28	25.1 2 ± 3.39	<0.001	25.07 ± 3.29	25.36 ± 3.48	0.023	25.07 ± 3.29	25.09 ± 3.29	0.414
Smoking, n (%)				0.008			<0.001			<0.001			<0.001			<0.001
Non-smoker	1,186,168 (55.93)	1,186,096 (55.93)	72 (52.17)		1,185,731 (55.93)	437 (50.23)		1,138,456 (55.39)	47,712 (72.63)		1,185,913 (55.93)	255 (38.81)		1,176,674 (56.00)	9494 (48.21)	
Former smoker	417,133 (19.67)	417,092 (19.67)	41 (29.71)		4,168,02 (19.66)	331 (38.05)		408,065 (19.86)	9068 (13.80)		416,951 (19.67)	182 (27.70)		412,319 (19.62)	4814 (24.45)	
Current smoker	517,599 (24.4)	517,574 (24.41)	25 (18.12)		517,497 (24.41)	102 (11.72)		508,683 (24.75)	8916 (13.57)		517,379 (24.40)	220 (33.49)		512,214 (24.38)	5385 (27.34)	
Alcohol consumption, n (%)				0.046			<0.001			<0.001			0.111			0.059
None	1,228,912 (57.94)	1,228,819 (57.94)	93 (67.39)		1,228,311 (57.94)	601 (69.08)		1,179,667 (57.40)	49,245 (74.96)		1,228,556 (57.94)	356 (54.19)		1,217,340 (57.94)	11,572 (58.76)	
Mild drinker	686,373 (32.36)	686,335 (32.36)	38 (27.54)		686,155 (32.37)	218 (25.06)		673,203 (32.76)	13,170 (20.05)		686,136 (32.36)	237 (36.07)		680,107 (32.37)	6266 (31.82)	
Heavy drinker	205,615 (9.69)	205,608 (9.70)	7 (5.07)		205,564 (9.70)	51 (5.86)		202,334 (9.84)	3281 (4.99)		205,551 (9.69)	64 (9.74)		203,760 (9.70)	1855 (9.42)	
Regular physical activity, n (%)	458,444 (21.62)	458,416 (21.62)	28 (20.29)	0.705	458,247 (21.62)	197 (22.64)	0.461	445,442 (21.67)	13,002 (19.79)	<0.001	458,324 (21.62)	120 (18.26)	0.037	454,077 (21.61)	4367 (22.18)	0.055
Low income, n (%)	492,625 (23.23)	492,595 (23.23)	30 (21.74)	0.679	492,461 (23.23)	164 (18.85)	0.0022	476,711 (23.20)	15,914 (24.22)	<0.001	492,491 (23.23)	134 (20.40)	0.086	487,903 (23.22)	4722 (23.98)	0.012
Rural residence, n (%)	1,182,177 (55.74)	1,182,096 (55.74)	81 (58.70)	0.484	1181725 (55.74)	452 (51.95)	0.025	1,143,587 (55.64)	38,590 (58.74)	<0.001	1,181,807 (55.74)	370 (56.32)	0.766	1,170,965 (55.73)	11,212 (56.93)	<0.001
Hypertension, n (%)	1,238,054 (58.37)	1,237,983 (58.37)	71 (51.45)	0.099	1,237,624 (58.38)	430 (49.43)	<0.001	1,195,358 (58.16)	42,696 (64.99)	<0.001	1,237,659 (58.37)	395 (60.12)	0.363	1,225,861 (58.34)	12,193 (61.92)	<0.001
Dyslipidemia, n (%)	912,871 (43.04)	912,829 (43.04)	42 (30.43)	0.003	912,522 (43.04)	349 (40.11)	0.081	878,388 (42.74)	34,483 (52.49)	<0.001	912,589 (43.04)	282 (42.92)	0.951	903,374 (42.99)	9497 (48.23)	<0.001
CKD, n (%)	242,950 (11.46)	242,929 (11.45)	21 (15.22)	0.165	242,863 (11.46)	87 (10.00)	0.178	232,954 (11.33)	9996 (15.22)	<0.001	242,888 (11.46)	62 (9.44)	0.104	240,512 (11.45)	2438 (12.38)	<0.001
Stroke, n (%)	103,532 (4.88)	103,525 (4.88)	7 (5.07)	0.917	103,490 (4.88)	42 (4.83)	0.941	98,274 (4.78)	5258 (8.00)	<0.001	103,505 (4.88)	27 (4.11)	0.358	102,231 (4.87)	1301 (6.61)	<0.001
Anemia, n (%)	62,933 (2.97)	62,921 (2.97)	12 (8.70)	<0.001	62,886 (2.97)	47 (5.40)	<0.001	58,900 (2.87)	4033 (6.14)	<0.001	62,903 (2.97)	30 (4.57)	0.016	62,221 (2.96)	712 (3.62)	<0.001
Osteoporosis, n (%)	196,123 (9.25)	196,103 (9.25)	20 (14.49)	0.033	196,022 (9.25)	101 (11.61)	0.016	178,075 (8.66)	18,048 (27.47)	<0.001	196,014 (9.24)	109 (16.59)	<0.001	194177 (9.24)	1946 (9.88)	0.002
Use of insulin, n (%)	194,571 (9.17)	194,546 (9.17)	25 (18.12)	<0.001	194,441 (9.17)	130 (14.94)	<0.001	184,032 (8.95)	10,539 (16.04)	<0.001	194,493 (9.17)	78 (11.87)	0.017	192,037 (9.14)	2534 (12.87)	<0.001
Number of oral hypoglycemic agents used 3, n (%)	323,566 (15.26)	323,551 (15.26)	15 (10.87)	0.152	323,443 (15.26)	123 (14.14)	0.359	311,205 (15.14)	12,361 (18.82)	<0.001	323,467 (15.26)	99 (15.07)	0.894	320,182 (15.24)	3384 (17.18)	<0.001
Duration of type 2 diabetes over 5 years, n (%)	676,322 (31.89)	676,287 (31.89)	35 (25.36)	0.100	676,043 (31.89)	279 (32.07)	0.909	652,060 (31.73)	24,262 (36.93)	<0.001	676,111 (31.89)	211 (32.12)	0.901	669,594 (31.87)	6728 (34.16)	<0.001

CD—Crohn’s disease; UC—ulcerative colitis; RA—rheumatoid arthritis; AS—ankylosing spondylitis; PsO—psoriasis; BMI—body mass index; CKD—chronic kidney disease; SD—standard deviation.

**Table 2:** Risk of overall fractures according to the presence of immune-mediated inflammatory diseases.

	No. of Participants	No. of Events	Total no. of Person-Years of Follow up	IR (Per 1000 Person-Years)	Model 1 HR (95% CI)	Model 2 HR (95% CI)	Model 3 HR (95% CI)	Model 4 HR (95% CI)	Model 5 HR (95% CI)	*p*
Overall fractures										
CD										
No	2,120,762	219,822	13,865,630.35	15.85	1 (Ref.)	1 (Ref.)	1 (Ref.)	1 (Ref.)	1 (Ref.)	0.398
Yes	138	17	890.21	19.10	1.23 (0.77, 1.97)	1.28 (0.80, 2.05)	1.30 (0.81, 2.09)	1.27 (0.79, 2.04)	1.28 (0.76, 1.97)	
UC										
No	2,120,030	219,725	13,860,881.72	15.85	1 (Ref.)	1 (Ref.)	1 (Ref.)	1 (Ref.)	1 (Ref.)	0.004
Yes	870	114	5638.84	20.22	1.28 (1.06, 1.53)	1.33 (1.10, 1.59)	1.36 (1.13, 1.64)	1.34 (1.12, 1.62)	1.31 (1.09, 1.57)	
RA										
No	2,055,204	209,451	13,454,973.84	15.57	1 (Ref.)	1 (Ref.)	1 (Ref.)	1 (Ref.)	1 (Ref.)	<0.001
Yes	65,696	10,388	411,546.72	25.24	1.63 (1.59, 1.66)	1.23 (1.21, 1.26)	1.24 (1.21, 1.26)	1.24 (1.21, 1.26)	1.19 (1.17, 1.22)	
AS										
No	2,120,243	219,775	13,862,299.67	15.85	1 (Ref.)	1 (Ref.)	1 (Ref.)	1 (Ref.)	1 (Ref.)	0.028
Yes	657	64	4220.90	15.16	0.96 (0.75, 1.22)	1.35 (1.06, 1.73)	1.36 (1.06, 1.73)	1.36 (1.07, 1.74)	1.32 (1.03, 1.68)	
PsO										
No	2,101,207	217,572	1,374,0471.17	15.83	1 (Ref.)	1 (Ref.)	1 (Ref.)	1 (Ref.)	1 (Ref.)	<0.001
Yes	19,693	2267	126,049.39	17.99	1.14 (1.09, 1.19)	1.16 (1.12, 1.2)	1.16 (1.11, 1.21)	1.16 (1.12, 1.21)	1.14 (1.10, 1.19)	
Number of IMIDs										
0	2,035,002	207,159	13,325,369.93	15.55	1 (Ref.)	1 (Ref.)	1 (Ref.)	1 (Ref.)	1 (Ref.)	<0.001
1	84,748	12,511	533,993.80	23.43	1.51 (1.48, 1.54)	1.22 (1.20, 1.25)	1.22 (1.20, 1.25)	1.22 (1.20, 1.25)	1.18 (1.16, 1.21)	
≥2	1150	169	7156.83	23.61	1.52 (1.31, 1.77)	1.36 (1.17, 1.58)	1.36 (1.17, 1.58)	1.36 (1.17, 1.58)	1.29 (1.11, 1.50)	

IR, incidence rate; HR, hazard ratio; CI, confidence interval; CD, Crohn’s disease; UC, ulcerative colitis; IMIDs, immune-mediated inflammatory diseases; RA, rheumatoid arthritis; AS, ankylosing spondylitis; PsO, psoriasis. Model 1 adjusted for none of the covariates (univariable analysis). Model 2 adjusted for age and sex. Model 3 adjusted for age, sex, smoking, alcohol consumption, regular physical activity. Model 4 adjusted for age, sex, smoking, alcohol consumption, regular physical activity, hypertension, dyslipidemia, and BMI. Model 5 adjusted for age, sex, smoking, alcohol consumption, regular physical activity, hypertension, dyslipidemia, BMI, depression, use of insulin, number of oral hypoglycemic agent ≥ 3, and duration of type 2 diabetes ≥ 5 years.

**Table 3:** Risk of osteoporotic fracture and non-osteoporotic fracture according to the presence of immune-mediated inflammatory diseases.

	No. of Participants	No. of Events	Total No. of Person-Years of Follow up	IR (Per 1000 Person-Years)	Model 1 HR (95% CI)	Model 2 HR (95% CI)	Model 3 HR (95% CI)	Model 4 HR (95% CI)	Model 5 HR (95% CI)	*p*
Osteoporotic fracture										
CD										
No	2,120,762	113,118	13,865,630.35	8.16	1 (Ref.)	1 (Ref.)	1 (Ref.)	1 (Ref.)	1 (Ref.)	0.193
Yes	138	11	890.21	12.36	1.52 (0.85, 2.75)	1.61 (0.89, 2.90)	1.61 (0.89, 2.91)	1.58 (0.87, 2.85)	1.48 (0.82, 2.67)	
UC										
No	2,120,030	113,072	13,860,881.72	8.16	1 (Ref.)	1 (Ref.)	1 (Ref.)	1 (Ref.)	1 (Ref.)	0.026
Yes	870	57	5638.84	10.11	1.24 (0.96, 1.61)	1.41 (1.09, 1.83)	1.42 (1.09, 1.84)	1.39 (1.08, 1.81)	1.34 (1.04, 1.74)	
RA										
No	2,055,204	105,778	13,454,973.84	7.86	1 (Ref.)	1 (Ref.)	1 (Ref.)	1 (Ref.)	1 (Ref.)	<0.001
Yes	65,696	7351	411,546.72	17.86	2.29 (2.23, 2.34)	1.45 (1.41, 1.48)	1.45 (1.41, 1.48)	1.45 (1.41, 1.48)	1.38 (1.35, 1.41)	
AS										
No	2,120,243	113,093	13,862,299.67	8.16	1 (Ref.)	1 (Ref.)	1 (Ref.)	1 (Ref.)	1 (Ref.)	<0.001
Yes	657	36	4220.90	8.53	1.05 (0.76, 1.45)	2.05 (1.48, 2.84)	2.04 (1.47, 2.83)	2.05 (1.48, 2.84)	1.95 (1.41, 2.70)	
PsO										
No	2,101,207	111,976	137,40471.17	8.15	1 (Ref.)	1 (Ref.)	1 (Ref.)	1 (Ref.)	1 (Ref.)	<0.001
Yes	19,693	1153	126,049.39	9.15	1.13 (1.06, 1.19)	1.25 (1.18, 1.32)	1.25 (1.18, 1.32)	1.25 (1.18, 1.32)	1.22 (1.15, 1.29)	
Number of IMIDs										
0	2,035,002	104,629	13,325,369.93	7.85	1 (Ref.)	1 (Ref.)	1 (Ref.)	1 (Ref.)	1 (Ref.)	<0.001
1	84,748	8392	533,993.80	15.72	2.01 (1.97, 2.06)	1.42 (1.39, 1.45)	1.42 (1.39, 1.45)	1.42 (1.39, 1.45)	1.36 (1.33, 1.39)	
≥2	1150	108	7156.83	15.09	1.94 (1.61, 2.34)	1.63 (1.35, 1.97)	1.63 (1.35, 1.97)	1.62 (1.34, 1.96)	1.54 (1.26, 1.84)	
Non-osteoporotic fracture										
CD										
No	2,120,762	106,704	13,865,630.35	7.70	1 (Ref.)	1 (Ref.)	1 (Ref.)	1 (Ref.)	1 (Ref.)	0.754
Yes	138	6	890.21	6.74	0.88 (0.40, 1.96)	0.89 (0.40, 1.98)	0.91 (0.41, 2.03)	0.89 (0.40, 1.98)	0.88 (0.40, 1.96)	
UC										
No	2,120,030	106,653	13,860,881.72	7.69	1 (Ref.)	1 (Ref.)	1 (Ref.)	1 (Ref.)	1 (Ref.)	0.052
Yes	870	57	5638.84	10.11	1.32 (1.01, 1.71)	1.29 (0.99, 1.67)	1.33 (1.02, 1.72)	1.32 (1.01, 1.71)	1.29 (1.00, 1.68)	
RA										
No	2,055,204	103,673	13,454,973.84	7.71	1 (Ref.)	1 (Ref.)	1 (Ref.)	1 (Ref.)	1 (Ref.)	<0.001
Yes	65696	3037	411,546.72	7.38	0.96 (0.92, 0.99)	0.93 (0.90, 0.97)	0.94 (0.90, 0.97)	0.94 (0.90, 0.97)	0.92 (0.88, 0.95)	
AS										
No	2,120,243	106,682	13,862,299.67	7.70	1 (Ref.)	1 (Ref.)	1 (Ref.)	1 (Ref.)	1 (Ref.)	0.534
Yes	657	28	4220.90	6.63	0.87 (0.60, 1.26)	0.90 (0.62, 1.30)	0.90 (0.62, 1.31)	0.91 (0.63, 1.31)	0.90 (0.61, 1.29)	
PsO										
No	2,101,207	105,596	13,740,471.17	7.69	1 (Ref.)	1 (Ref.)	1 (Ref.)	1 (Ref.)	1 (Ref.)	<0.001
Yes	19,693	1114	126,049.39	8.84	1.15 (1.08, 1.22)	1.12 (1.06, 1.19)	1.12 (1.06, 1.19)	1.13 (1.06, 1.20)	1.11 (1.05, 1.18)	
Number of IMIDs										
0	2,035,002	102,530	13,325,369.93	7.69	1 (Ref.)	1 (Ref.)	1 (Ref.)	1 (Ref.)	1 (Ref.)	0.050
1	84,748	4119	533,993.80	7.71	1 (0.97, 1.03)	0.98 (0.95, 1.01)	0.98 (0.95, 1.01)	0.98 (0.95, 1.01)	0.96 (0.93, 0.99)	
≥2	1150	61	7156.83	8.52	1.11 (0.86, 1.43)	1.08 (0.84, 1.39)	1.09 (0.85, 1.40)	1.09 (0.85, 1.40)	1.06 (0.82, 1.36)	

IR—incidence rate; HR—hazard ratio; CI—confidence interval; CD—Crohn’s disease; UC—ulcerative colitis; IMIDs—immune-mediated inflammatory diseases; RA—rheumatoid arthritis; AS—ankylosing spondylitis; PsO—psoriasis. Model 1 adjusted for none of the covariates (univariable analysis). Model 2 adjusted for age and sex. Model 3 adjusted for age, sex, smoking, alcohol consumption, and regular physical activity. Model 4 adjusted for age, sex, smoking, alcohol consumption, regular physical activity, hypertension, dyslipidemia, and BMI. Model 5 adjusted for age, sex, smoking, alcohol consumption, regular physical activity, hypertension, dyslipidemia, BMI, depression, use of insulin, number of oral hypoglycemic agent ≥ 3, and duration of type 2 diabetes ≥ 5 years.

**Table 4:** Risk of vertebral fracture and hip fracture according to the presence of immune-mediated inflammatory diseases.

	No. of Participants	No. of Events	Total No. of Person-Years of Follow up	IR (per 1000 Person-Years)	Model 1 HR (95% CI)	Model 2 HR (95% CI)	Model 3 HR (95% CI)	Model 4 HR (95% CI)	Model 5 HR (95% CI)	*p*
Vertebral fracture										
CD										
No	2,120,762	65,675	14,416,786.45	4.56	1 (Ref.)	1 (Ref.)	1 (Ref.)	1 (Ref.)	1 (Ref.)	0.531
Yes	138	3	931.72	3.22	0.75 (0.25, 2.24)	0.74 (0.24,2.28)	0.74 (0.24, 2.29)	0.73 (0.24, 2.27)	0.70 (0.23, 2.16)	
UC										
No	2,120,030	65,642	1,441,1796.77	4.55	1 (Ref.)	1 (Ref.)	1 (Ref.)	1 (Ref.)	1 (Ref.)	0.047
Yes	870	36	5921.40	6.08	1.33 (0.96, 1.85)	1.42 (1.02, 1.97)	1.44 (1.04, 2.00)	1.43 (1.03, 1.99)	1.39 (1.00, 1.93)	
RA										
No	2,055,204	61,953	13,982,052.26	4.43	1 (Ref.)	1 (Ref.)	1 (Ref.)	1 (Ref.)	1 (Ref.)	<0.001
Yes	65,696	3725	435,665.90	8.55	1.93 (1.87, 2.00)	1.35 (1.31, 1.39)	1.35 (1.31, 1.40)	1.35 (1.31, 1.40)	1.30 (1.26, 1.34)	
AS										
No	2,120,243	65,652	14,413,340.35	4.55	1 (Ref.)	1 (Ref.)	1 (Ref.)	1 (Ref.)	1 (Ref.)	0.001
Yes	657	26	4377.81	5.94	1.31 (0.89, 1.92)	2.19 (1.49, 3.21)	2.18 (1.49, 3.21)	2.19 (1.49, 3.21)	2.11 (1.44, 3.11)	
PsO										
No	2,101,207	64,926	14,286,224.00	4.54	1 (Ref.)	1 (Ref.)	1 (Ref.)	1 (Ref.)	1 (Ref.)	<0.001
Yes	19,693	752	131,494.16	5.72	1.26 (1.17, 1.36)	1.31 (1.22, 1.41)	1.31 (1.22, 1.41)	1.31 (1.22, 1.41)	1.29 (1.20, 1.38)	
Number of IMIDs										
0	2,035,002	61,197	13,846,883.08	4.42	1 (Ref.)	1 (Ref.)	1 (Ref.)	1 (Ref.)	1 (Ref.)	<0.001
1	84,748	4420	563,318.69	7.85	1.78 (1.73, 1.83)	1.35 (1.31, 1.39)	1.35 (1.31, 1.39)	1.35 (1.31, 1.39)	1.30 (1.26, 1.34)	
≥2	1150	61	7516.40	8.12	1.85 (1.44, 2.37)	1.60 (1.24, 2.05)	1.59 (1.24, 2.05)	1.59 (1.24, 2.05)	1.51 (1.18, 1.94)	
Hip fracture										
CD										
No	2,120,762	26,086	14,566,878.07	1.79	1 (Ref.)	1 (Ref.)	1 (Ref.)	1 (Ref.)	1 (Ref.)	0.316
Yes	138	3	939.61	3.19	1.81 (0.59, 5.57)	2.01 (0.65, 6.22)	2.04 (0.66, 6.34)	1.83 (0.59, 5.66)	1.78 (0.58, 5.53)	
UC										
No	2,120,030	26,077	14,561,834.62	1.79	1 (Ref.)	1 (Ref.)	1 (Ref.)	1 (Ref.)	1 (Ref.)	0.687
Yes	870	12	5983.06	2.01	1.12 (0.64, 1.98)	1.17 (0.66, 2.05)	1.22 (0.69, 2.14)	1.18 (0.67, 2.08)	1.12 (0.64, 1.98)	
RA										
No	2,055,204	24,684	14,123,511.73	1.75	1 (Ref.)	1 (Ref.)	1 (Ref.)	1 (Ref.)	1 (Ref.)	<0.001
Yes	65,696	1405	444,305.95	3.16	1.82 (1.72, 1.92)	1.26 (1.20, 1.33)	1.27 (1.20, 1.34)	1.27 (1.21, 1.34)	1.19 (1.13, 1.26)	
AS										
No	2,120,243	26,080	14,563,367.88	1.79	1 (Ref.)	1 (Ref.)	1 (Ref.)	1 (Ref.)	1 (Ref.)	0.030
Yes	657	9	4449.80	2.02	1.14 (0.60, 2.19)	2.11 (1.10, 4.05)	2.11 (1.10, 4.05)	2.14 (1.12, 4.12)	2.06 (1.07, 3.96)	
PsO										
No	2,101,207	25,801	14,434,581.39	1.79	1 (Ref.)	1 (Ref.)	1 (Ref.)	1 (Ref.)	1 (Ref.)	0.011
Yes	19,693	288	133,236.29	2.16	1.21 (1.08, 1.36)	1.21 (1.08, 1.36)	1.20 (1.07, 1.35)	1.21 (1.08, 1.36)	1.16 (1.03, 1.31)	
Number of IMIDs										
0	2,035,002	24,395	13,986,649.78	1.74	1 (Ref.)	1 (Ref.)	1 (Ref.)	1 (Ref.)	1 (Ref.)	<0.001
1	84,748	1672	573,459.70	2.92	1.68 (1.60, 1.76)	1.26 (1.20, 1.32)	1.26 (1.20, 1.33)	1.27 (1.21, 1.33)	1.19 (1.13, 1.25)	
≥2	1150	22	7708.20	2.85	1.65 (1.09, 2.50)	1.43 (0.94, 2.17)	1.41 (0.93, 2.14)	1.41 (0.93, 2.14)	1.31 (0.86, 1.98)	

IR—incidence rate; HR—hazard ratio; CI—confidence interval; CD—Crohn’s disease; UC—ulcerative colitis; IMIDs—immune-mediated inflammatory diseases; RA—rheumatoid arthritis; AS—ankylosing spondylitis; PsO—psoriasis. Model 1 adjusted for none of the covariates (univariable analysis). Model 2 adjusted for age and sex. Model 3 adjusted for age, sex, smoking, alcohol consumption, and regular physical activity. Model 4 adjusted for age, sex, smoking, alcohol consumption, regular physical activity, hypertension, dyslipidemia, and BMI. Model 5 adjusted for age, sex, smoking, alcohol consumption, regular physical activity, hypertension, dyslipidemia, BMI, depression, use of insulin, number of oral hypoglycemic agent ≥ 3, and duration of type 2 diabetes ≥ 5 years.

## Data Availability

Data are contained within the article and Supplementary Materials.

## Supplementary Materials

The following supporting information can be downloaded at: https://www.mdpi.com/article/10.3390/jcm14030795/s1, Figure S1. Cumulative incidence of osteoporotic fractures according to the presence of each IMID and the number of comorbid IMIDs; Figure S2. Cumulative incidence of non-osteoporotic fractures according to the presence of each IMID and the number of comorbid IMIDs; Figure S3. Cumulative incidece of vertebral fractures according to the presence of each IMID and the number of comorbid IMIDs; Figure S4. Cumulative incidence of hip fractures according to the presence of each IMID and the number of comorbid IMIDs; Table S1. Subgroup analysis of the risk for overall fractures according to the number of IMIDs; Table S2. Subgroup analysis of the risk for overall fractures according to the presence of psoriasis and rheumatoid arthritis; Table S3. Subgroup analysis of the risk for osteoporotic fractures according to the presence of psoriasis and rheumatoid arthritis; Table S4. Subgroup analysis of the risk for non-osteoporotic fractures according to the presence of psoriasis and rheumatoid arthritis; Table S5. Subgroup analysis of the risk for vertebral fractures according to the presence of psoriasis and rheumatoid arthritis; Table S6. Subgroup analysis of the risk for hip fractures according to the presence of psoriasis and rheumatoid arthritis.
