# Supplementary material for: Association of Immune-Mediated Inflammatory Diseases and Fracture Risk in Patients with Type 2 Diabetes: A Nationwide Population-Based Study
Source: J Clin Med. 2025 Jan 25;14(3):795. doi: 10.3390/jcm14030795 (PMC11818108; doi:10.3390/jcm14030795)
Supplement: Supplementary file 1 [file jcm-14-00795-s001.zip › jcm-3353939-supplementary.pdf]

Supplement materials

Methods.

Definition of covariates and outcomes

CD was defined as ICD-10 code K50 with RID code V130.[1,2] UC was defined as ICD-10 code K51 with RID code V131.[1,2] RA was defined as ICD-10 code M05 or M06 with prescriptions for disease-modifying antirheumatic drugs.[3,4] AS was defined as ICD-10 code M45 with RID code V140,[5] and PsO was defined as ICD-10 code L40.[6] We obtained data on the following comorbidities: hypertension was defined as ICD 10 codes I10–I13 and I15 with prescriptions for antihypertensive agents, or systolic or diastolic blood pressure ≥140 or ≥90 mmHg, respectively. Dyslipidemia was defined as ICD-10 code E78 with prescriptions for lipid-lowering agents or serum total cholesterol ≥240 mg/dL. Stroke was defined as ICD-10 codes I63 or I64 during hospitalization with claims for brain magnetic resonance imaging or computed tomography. Chronic kidney disease was defined as an estimated glomerular filtration rate <60 mL/min/1.73 m^2.[7] Anemia was defined as ICD-10 code D64.9 or hemoglobin level <14 g/dL for males and <13 g/dL for females. Osteoporosis was defined as ICD-codes M80-82.[8-18]

Osteoporotic fractures were defined as fractures in four specific parts: spine (ICD-10 codes S22.0, S22.1, S32.0, S32.7, M48.4, and M48.5), proximal humerus (ICD-10 codes S422 and S423), femur (ICD-10 code S72), and distal radius (ICD-10 codes S525 and S526), with a diagnosis of osteoporosis before or within 90 days of the fracture.[19] Non-osteoporotic fractures were defined as overall fractures excluding osteoporotic fractures. Hip fractures were defined using ICD-10 codes S72.0 and S72.1, and vertebral fractures were defined using ICD-10 codes S22.0, S22.1, S32.0, M48.4, and M48.5.[20] DM duration was defined as (the date of health check-up – the date of first diabetes medication claim)/365.

References.

1. Kang EA, Chun J, Kim JH, et al. Periodontitis combined with smoking increases risk of the ulcerative colitis: A national cohort study. World J Gastroenterol. Oct 7 2020;26(37):5661-5672. doi:10.3748/wjg.v26.i37.5661
2. Kim J, Chun J, Lee C, et al. Increased risk of idiopathic pulmonary fibrosis in inflammatory bowel disease: A nationwide study. J Gastroenterol Hepatol. Feb 2020;35(2):249-255. doi:10.1111/jgh.14838
3. Kim SY, Servi A, Polinski JM, et al. Validation of rheumatoid arthritis diagnoses in health care utilization data. Arthritis Res Ther. Feb 23 2011;13(1):R32. doi:10.1186/ar3260
4. Lee EE, Shin A, Lee J, et al. All-cause and cause-specific mortality of patients with rheumatoid arthritis in Korea: A nation-wide population-based study. Joint Bone Spine. Jan 2022;89(1):105269. doi:10.1016/j.jbspin.2021.105269
5. Lee JS, Oh JS, Kim YJ, et al. Reasons for the High Cesarean Delivery Rate among Women with Ankylosing Spondylitis: Using the Korean National Health Insurance Database. J Rheumatol. May 1 2020;47(5):668-673. doi:10.3899/jrheum.190754
6. Han JH, Lee JH, Han KD, et al. Epidemiology and Medication Trends in Patients with Psoriasis: A

Nationwide Population-based Cohort Study from Korea. Acta Derm Venereol. Apr 16 2018;98(4):396-400. doi:10.2340/00015555-2877
7. Levey AS, Bosch JP, Lewis JB, Greene T, Rogers N, Roth D. A more accurate method to estimate glomerular filtration rate from serum creatinine: a new prediction equation. Modification of Diet in Renal Disease Study Group. Ann Intern Med. Mar 16 1999;130(6):461-70. doi:10.7326/0003-4819-130-6-199903160-00002
8. Hong S, Park JH, Han K, Lee CB, Kim DS, Yu SH. Association Between Obesity and Cardiovascular Disease in Elderly Patients With Diabetes: A Retrospective Cohort Study. J Clin Endocrinol Metab. Jan 18 2022;107(2):e515-e527. doi:10.1210/clinem/dgab714
9. Nam GE, Kim W, Han K, et al. Body Weight Variability and the Risk of Cardiovascular Outcomes and Mortality in Patients With Type 2 Diabetes: A Nationwide Cohort Study. Diabetes Care. Sep 2020;43(9):2234-2241. doi:10.2337/dc19-2552
10. Kang YM, Cho YK, Lee SE, et al. Cardiovascular Diseases and Life Expectancy in Adults With Type 2 Diabetes: A Korean National Sample Cohort Study. J Clin Endocrinol Metab. Sep 1 2017;102(9):3443-3451. doi:10.1210/jc.2017-00643
11. Thuluvath PJ, Alukal JJ, Zhang T. Impact of Hyponatremia on Morbidity, Mortality, and Resource Utilization in Portal Hypertensive Ascites: A Nationwide Analysis. J Clin Exp Hepatol. May-Jun 2022;12(3):871-875. doi:10.1016/j.jceh.2021.10.145
12. Park S, Chun J, Han KD, et al. Increased end-stage renal disease risk in patients with inflammatory bowel disease: A nationwide population-based study. World J Gastroenterol. Nov 14 2018;24(42):4798-4808. doi:10.3748/wjg.v24.i42.4798
13. Lee SR, Choi EK, Rhee TM, et al. Evaluation of the association between diabetic retinopathy and the incidence of atrial fibrillation: A nationwide population-based study. Int J Cardiol. Nov 15 2016;223:953-957. doi:10.1016/j.ijcard.2016.08.296
14. Soh H, Chun J, Han K, et al. Increased Risk of Herpes Zoster in Young and Metabolically Healthy Patients with Inflammatory Bowel Disease: A Nationwide Population-Based Study. Gut Liver. May 15 2019;13(3):333-341. doi:10.5009/gnl18304
15. Kim HY, Jang EJ, Park B, et al. Development of a Korean Fracture Risk Score (KFRS) for Predicting Osteoporotic Fracture Risk: Analysis of Data from the Korean National Health Insurance Service. PLoS One. 2016;11(7):e0158918. doi:10.1371/journal.pone.0158918
16. Park J-H, Lee J, Yu S-Y, et al. Comparing proton pump inhibitors with histamin-2 receptor blockers regarding the risk of osteoporotic fractures: a nested case-control study of more than 350,000 Korean patients with GERD and peptic ulcer disease. BMC Geriatrics. 2020/10/15 2020;20(1):407. doi:10.1186/s12877-020-01794-3
17. Kang SH, Moon S-J, Kang M, Chung SJ, Cho GJ, Koh S-B. Incidence of Parkinson's disease and modifiable risk factors in Korean population: A longitudinal follow-up study of a nationwide cohort. Original Research. Frontiers in Aging Neuroscience. 2023-February-14 2023;15doi:10.3389/fnagi.2023.1094778
18. Kwon MJ, Park JY, Kim SG, et al. Potential Association of Osteoporosis and Not Osteoporotic Fractures in Patients with Gout: A Longitudinal Follow-Up Study. Nutrients. Dec 28 2022;15(1)doi:10.3390/nu15010134
19. Ahn SH, Park SM, Park SY, et al. Osteoporosis and Osteoporotic Fracture Fact Sheet in Korea. J Bone Metab. Nov 2020;27(4):281-290. doi:10.11005/jbm.2020.27.4.281

20. Kang DW, Wang SM, Um YH, et al. Differential Risk of Incident Fractures Depending on Intensity and Frequency of Physical Activity According to Cognitive Status: A Nationwide Longitudinal Study. Front Med (Lausanne). 2020;7:572466. doi:10.3389/fmed.2020.572466

Figure S1. Cumulative incidence of osteoporotic fractures according to the presence of each IMID and the number of comorbid IMIDs. CD, Crohn's disease; UC, ulcerative colitis; RA, rheumatoid arthritis; AS, ankylosing spondylitis; PsO, psoriasis; IMIDs, immune-mediated inflammatory disease.

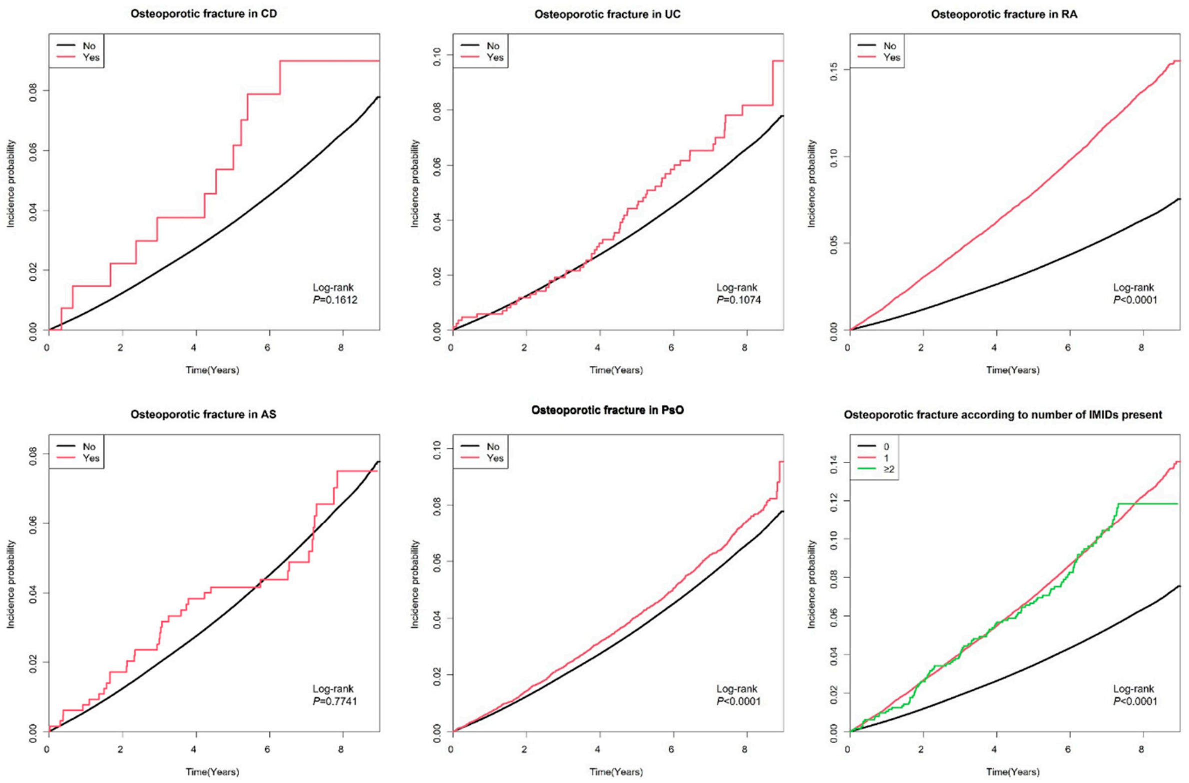

Figure S2. Cumulative incidence of non-osteoporotic fractures according to the presence of each IMID and the number of comorbid IMIDs. CD, Crohn's disease; UC, ulcerative colitis; RA, rheumatoid arthritis; AS, ankylosing spondylitis; PsO, psoriasis; IMIDs, immune-mediated inflammatory disease.

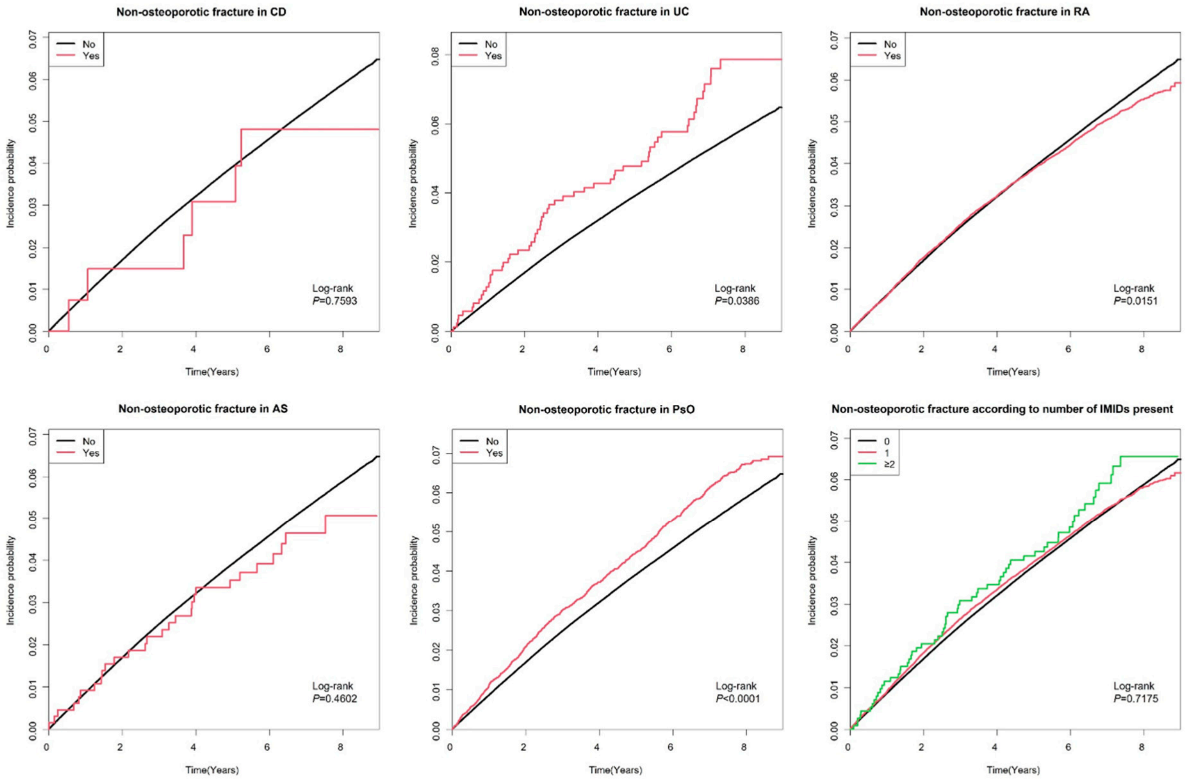

Figure S3. Cumulative incidence of vertebral fractures according to the presence of each IMID and the number of comorbid IMIDs. CD, Crohn's disease; UC, ulcerative colitis; RA, rheumatoid arthritis; AS, ankylosing spondylitis; PsO, psoriasis; IMIDs, immune-mediated inflammatory disease.

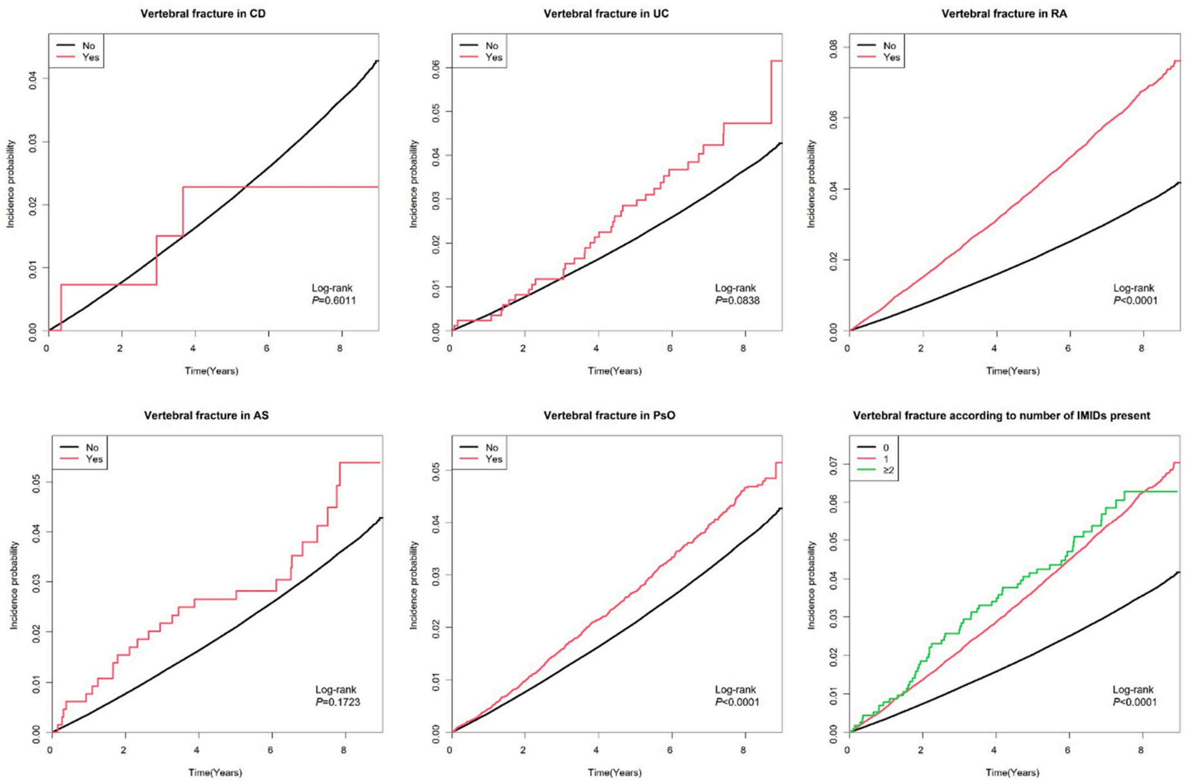

Figure S4. Cumulative incidence of hip fractures according to the presence of each IMID and the number of comorbid IMIDs. CD, Crohn's disease; UC, ulcerative colitis; RA, rheumatoid arthritis; AS, ankylosing spondylitis; PsO, psoriasis; IMIDs, immune-mediated inflammatory disease.

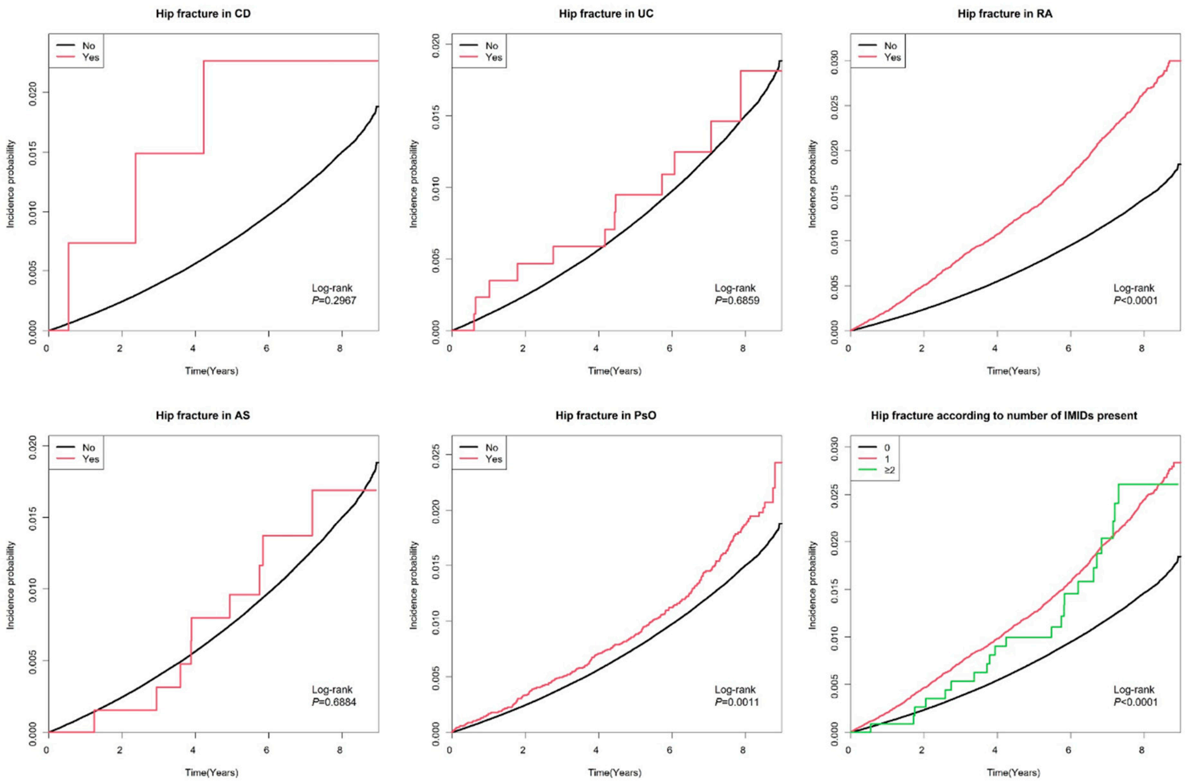

Table S1. Subgroup analysis of the risk for overall fractures according to the number of IMIDs

Subgroup	Number of IMID	No. of participants	No. of events	Total no. of person-years of follow up	IR (per 1,000 person-years)	Model 5 HR (95% CI)	*P for interaction*
Age							0.499
<65	0	1481370	114361	9968085.55	11.47	1 (Ref.)	
	1	14352	1288	94936.17	13.57	1.18 (1.12, 1.25)	
	≥2	21	2	145.12	13.78	1.34 (0.34, 5.35)	
≥65	0	618210	103022	3761876.36	27.39	1 (Ref.)	
	1	6930	1162	41381.80	28.08	1.13 (1.06, 1.19)	
	≥2	17	4	95.57	41.85	1.53 (0.58, 4.06)	
Sex							0.438
Male	0	1266604	88323	8356050.08	10.57	1 (Ref.)	
	1	14550	1280	93640.19	13.67	1.18 (1.12, 1.25)	
	≥2	27	3	177.27	16.92	1.40 (0.45, 4.35)	
Female	0	832976	129060	5373911.82	24.02	1 (Ref.)	
	1	6732	1170	42677.78	27.41	1.12 (1.06, 1.19)	
	≥2	11	3	63.42	47.31	1.56 (0.51, 4.77)	
Smoking							0.956
Non-smoker	0	1175928	151325	7653983.72	19.77	1 (Ref.)	
	1	10222	1471	64942.13	22.65	1.16 (1.10, 1.22)	
	≥2	18	4	98.78	40.50	1.80 (0.67, 4.78)	
Former smoker	0	411778	28913	2724958.36	10.61	1 (Ref.)	
	1	5342	450	34758.17	12.95	1.13 (1.03, 1.24)	
	≥2	13	2	89.42	22.37	1.69 (0.42, 6.77)	
Current	0	511874	37145	3351019.82	11.08	1 (Ref.)	
	1	5718	529	36617.67	14.45	1.17 (1.07, 1.28)	
	≥2	7	0	52.49	0	-	
Alcohol consumption							0.892
Non	0	1216315	153567	7850745.32	19.56	1 (Ref.)	
	1	12572	1683	79070.15	21.28	1.14 (1.09, 1.20)	
	≥2	25	6	154.53	38.83	1.83 (0.82, 4.07)	
Mild	0	679624	48447	4536118.78	10.68	1 (Ref.)	
	1	6739	585	44283.38	13.21	1.19 (1.09, 1.29)	
	≥2	10	0	66.93	0	-	
Heavy	0	203641	15369	1343097.80	11.44	1 (Ref.)	
	1	1971	182	12964.45	14.04	1.17 (1.01, 1.35)	
	≥2	3	0	19.23	0	-	
Regular physical activity							0.150
No	0	1645839	173938	10703926.91	16.25	1 (Ref.)	
	1	16588	1976	105442.47	18.74	1.18 (1.13, 1.23)	
	≥2	29	5	176.67	28.30	1.63 (0.68, 3.90)	
Yes	0	453741	43445	3026034.99	14.36	1 (Ref.)	
	1	4694	474	30875.50	15.35	1.07 (0.97, 1.17)	
	≥2	9	1	64.01	15.62	0.96 (0.14, 6.84)	
Duration of type 2 diabetes							0.537
<5 years	0	1430493	129397	9410354.97	13.75	1 (Ref.)	
	1	14065	1412	91049.63	15.51	1.15 (1.09, 1.21)	
	≥2	20	2	132.61	15.08	0.88 (0.22, 3.53)	
≥5 years	0	669087	87986	4319606.93	20.37	1 (Ref.)	
	1	7217	1038	45268.34	22.93	1.16 (1.10, 1.24)	
	≥2	18	4	108.08	37.01	2.17 (0.82, 5.78)	

IR, incidence rate; HR, hazard ratio; CI, confidence interval

Model 5 adjusted for age, sex, smoking, alcohol consumption, regular physical activity, hypertension, dyslipidemia, BMI, depression, use of insulin, number of oral hypoglycemic agent ≥ 3, and duration of type 2 diabetes ≥ 5 years

The P values listed in the tables represent the interaction between the subgroups.

Table S2. Subgroup analysis of the risk for overall fractures according to the presence of psoriasis and rheumatoid arthritis

Subgroup	PsO	No. of participants	No. of events	Total no. of person-years of follow up	IR (per 1,000 person-years)	Model 5 HR (95% CI)	*P for interaction*
Age							0.312
<65	No	1482567	114468	9975959.89	11.47	1(Ref.)	
	Yes	13176	1183	87206.94	13.57	1.17 (1.10, 1.24)	
≥65	No	618640	103104	3764511.27	27.39	1(Ref.)	
	Yes	6517	1084	38842.46	27.91	1.12 (1.05, 1.20)	
Sex							0.175
Male	No	1267810	88424	8363908.56	10.57	1(Ref.)	
	Yes	13371	1182	85958.98	13.75	1.18 (1.11, 1.24)	
Female	No	833397	129148	5376562.60	24.02	1(Ref.)	
	Yes	6322	1085	40090.42	27.06	1.11 (1.05, 1.18)	
Smoking							0.924
Non-smoker	No	1176674	151440	7658722.13	19.77	1(Ref.)	
	Yes	9494	1360	60302.50	22.55	1.15 (1.09, 1.21)	
Former smoker	No	412319	28956	2728610.04	10.61	1(Ref.)	
	Yes	4814	409	31195.91	13.11	1.12 (1.02, 1.24)	
Current	No	512214	37176	3353139.00	11.09	1(Ref.)	
	Yes	5385	498	34550.98	14.41	1.15 (1.06, 1.26)	
Alcohol consumption							0.705
Non	No	1217340	153705	7857287.64	19.56	1(Ref.)	
	Yes	11572	1551	72682.36	21.34	1.13 (1.07, 1.19)	
Mild	No	680107	48489	4539300.77	10.68	1(Ref.)	
	Yes	6266	543	41168.31	13.19	1.17 (1.08, 1.28)	
Heavy	No	203760	15378	1343882.76	11.44	1(Ref.)	
	Yes	1855	173	12198.72	14.18	1.17 (1.01, 1.36)	
Regular physical activity							0.041
No	No	1647130	174087	10712227.02	16.25	1(Ref.)	
	Yes	15326	1832	97319.03	18.82	1.17 (1.12, 1.22)	
Yes	No	454077	43485	3028244.15	14.36	1(Ref.)	
	Yes	4367	435	28730.36	15.14	1.05 (0.95, 1.15)	
Duration of type 2 diabetes							0.518
<5 years	No	1431613	129515	9417602.75	13.75	1(Ref.)	
	Yes	12965	1296	83934.45	15.44	1.13 (1.07, 1.19)	
≥5 years	No	669594	88057	4322868.41	20.37	1(Ref.)	
	Yes	6728	971	42114.94	23.06	1.16 (1.09, 1.24)	
	RA						
Age							0.097
<65	No	1456938	110992	9809317.33	11.31	1 (Ref.)	
	Yes	38805	4659	253849.50	18.35	1.21 (1.18, 1.25)	
≥65	No	598266	98459	3645656.51	27.01	1 (Ref.)	
	Yes	26891	5729	157697.22	36.33	1.17 (1.14, 1.21)	
Sex							<.001
Male	No	1257075	87197	8298754.20	10.51	1 (Ref.)	
	Yes	24106	2409	151113.35	15.94	1.27 (1.22, 1.32)	
Female	No	798129	122254	5156219.64	23.71	1 (Ref.)	
	Yes	41590	7979	260433.38	30.64	1.17 (1.14, 1.20)	
Smoking							0.020
Non-smoker	No	1138456	144358	7419864.91	19.46	1 (Ref.)	
	Yes	47712	8442	299159.72	28.22	1.18 (1.15, 1.20)	
Former smoker	No	408065	28422	2702638.90	10.52	1 (Ref.)	
	Yes	9068	943	57167.05	16.50	1.27 (1.20, 1.363)	
Current	No	508683	36671	3332470.03	11.00	1 (Ref.)	
	Yes	8916	1003	55219.96	18.16	1.25 (1.17, 1.33)	
Alcohol consumption							0.587
Non	No	1179667	146633	7624205.14	19.23	1 (Ref.)	
	Yes	49245	8623	305764.86	28.20	1.19 (1.16, 1.21)	
Mild	No	673203	47604	4495533.04	10.59	1 (Ref.)	
	Yes	13170	1428	84936.04	16.81	1.22 (1.16, 1.29)	
Heavy	No	202334	15214	1335235.65	11.39	1 (Ref.)	
	Yes	3281	337	20845.83	16.17	1.20 (1.08, 1.34)	
Regular physical activity							0.462
No	No	1609762	167364	10481565.51	15.97	1 (Ref.)	

	Yes	52694	8555	327980.55	26.08	1.20 (1.17, 1.22)	
Yes	No	445442	42087	2973408.33	14.15	1 (Ref.)	
	Yes	13002	1833	83566.18	21.93	1.17 (1.12, 1.23)	
Duration of type 2 diabetes							0.003
<5 years	No	1403144	124840	9240373.83	13.51	1 (Ref.)	
	Yes	41434	5971	261163.38	22.86	1.22 (1.19, 1.25)	
≥5 years	No	652060	84611	4214600.01	20.08	1 (Ref.)	
	Yes	24262	4417	150383.35	29.37	1.15 (1.12, 1.19)	

PsO, psoriasis; RA, rheumatoid arthritis; IR, incidence rate; HR, hazard ratio; CI, confidence interval
Model 5 adjusted for age, sex, smoking, alcohol consumption, regular physical activity, hypertension, dyslipidemia, BMI, depression, use of insulin, number of oral hypoglycemic agent ≥ 3, and duration of type 2 diabetes ≥ 5 years
The P values listed in the tables represent the interaction between the subgroups.

Table S3. Subgroup analysis of the risk for osteoporotic fractures according to the presence of psoriasis and rheumatoid arthritis

Subgroup	PsO	No. of participants	No. of events	Total no. of person-years of follow up	IR (per 1,000 person-years)	Model 5 HR (95% CI)	*P for interaction*
Age							0.843
<65	No	1482567	41199	9975959.89	4.13	1 (Ref.)	
	Yes	13176	431	87206.94	4.94	1.23 (1.12, 1.35)	
≥65	No	618640	70777	3764511.27	18.80	1 (Ref.)	
	Yes	6517	722	38842.46	18.59	1.21 (1.13, 1.30)	
Sex							0.005
Male	No	1267810	23120	8363908.56	2.76	1 (Ref.)	
	Yes	13371	385	85958.98	4.48	1.37 (1.24, 1.52)	
Female	No	833397	88856	5376562.60	16.53	1 (Ref.)	
	Yes	6322	768	40090.42	19.16	1.15 (1.07, 1.24)	
Smoking							0.104
Non-smoker	No	1176674	93780	7658722.13	12.24	1 (Ref.)	
	Yes	9494	838	60302.50	13.90	1.18 (1.10, 1.26)	
Former smoker	No	412319	8454	2728610.04	3.10	1 (Ref.)	
	Yes	4814	159	31195.91	5.10	1.42 (1.21, 1.66)	
Current	No	512214	9742	3353139.00	2.91	1 (Ref.)	
	Yes	5385	156	34550.98	4.52	1.25 (1.07,1.46)	
Alcohol consumption							0.355
Non	No	1217340	94090	7857287.64	11.97	1 (Ref.)	
	Yes	11572	932	72682.36	12.82	1.19 (1.12, 1.27)	
Mild	No	680107	14654	4539300.77	3.23	1 (Ref.)	
	Yes	6266	181	41168.31	4.40	1.34 (1.16, 1.55)	
Heavy	No	203760	3232	1343882.76	2.41	1 (Ref.)	
	Yes	1855	40	12198.72	3.28	1.23 (0.90, 1.68)	
Regular physical activity							0.029
No	No	1647130	91665	10712227.02	8.56	1 (Ref.)	
	Yes	15326	964	97319.03	9.91	1.25 (1.18, 1.34)	
Yes	No	454077	20311	3028244.15	6.71	1 (Ref.)	
	Yes	4367	189	28730.36	6.58	1.05 (0.91, 1.22)	
Duration of type 2 diabetes							0.101
<5 years	No	1431613	62623	9417602.75	6.65	1 (Ref.)	
	Yes	12965	599	83934.45	7.14	1.16 (1.07, 1.26)	
≥5 years	No	669594	49353	4322868.41	11.42	1 (Ref.)	
	Yes	6728	554	42114.94	13.15	1.28 (1.18, 1.39)	
	RA						
Age							<.001
<65	No	1456938	38955	9809317.33	3.97	1 (Ref.)	
	Yes	38805	2675	253849.50	10.54	1.58 (1.52, 1.64)	
≥65	No	598266	66823	3645656.51	18.33	1 (Ref.)	
	Yes	26891	4676	157697.22	29.65	1.28 (1.24, 1.32)	
Sex							<.001
Male	No	1257075	22489	8298754.20	2.71	1 (Ref.)	
	Yes	24106	1016	151113.35	6.72	1.86 (1.75, 1.98)	
Female	No	798129	83289	5156219.64	16.15	1 (Ref.)	
	Yes	41590	6335	260433.38	24.32	1.32 (1.29, 1.35)	
Smoking							<.001
Non-smoker	No	1138456	88169	7419864.91	11.88	1 (Ref.)	
	Yes	47712	6449	299159.72	21.56	1.34 (1.30, 1.37)	
Former smoker	No	408065	8182	2702638.90	3.03	1 (Ref.)	
	Yes	9068	431	57167.05	7.54	1.72 (1.56, 1.90)	
Current	No	508683	9427	3332470.03	2.83	1 (Ref.)	
	Yes	8916	471	55219.96	8.53	1.75 (1.59, 1.92)	
Alcohol consumption							<.001
Non	No	1179667	88498	7624205.14	11.61	1 (Ref.)	
	Yes	49245	6524	305764.86	21.34	1.35 (1.31, 1.38)	
Mild	No	673203	14133	4495533.04	3.14	1 (Ref.)	
	Yes	13170	702	84936.04	8.27	1.61 (1.49, 1.73)	
Heavy	No	202334	3147	1335235.65	2.36	1 (Ref.)	
	Yes	3281	125	20845.83	6.00	1.87 (1.57, 2.24)	
Regular physical activity							0.310
No	No	1609762	86522	10481565.51	8.25	1 (Ref.)	

	Yes	52694	6107	327980.55	18.62	1.37 (1.34, 1.41)	
Yes	No	445442	19256	2973408.33	6.48	1 (Ref.)	
	Yes	13002	1244	83566.18	14.89	1.42 (1.34, 1.5)	
Duration of type 2 diabetes							<.001
<5 years	No	1403144	59017	9240373.83	6.39	1 (Ref.)	
	Yes	41434	4205	261163.38	16.10	1.46 (1.42, 1.51)	
≥5 years	No	652060	46761	4214600.01	11.10	1 (Ref.)	
	Yes	24262	3146	150383.35	20.92	1.28 (1.23, 1.33)	

PsO, psoriasis; RA, rheumatoid arthritis; IR, incidence rate; HR, hazard ratio; CI, confidence interval

Model 5 adjusted for age, sex, smoking, alcohol consumption, regular physical activity, hypertension, dyslipidemia, BMI, depression, use of insulin, number of oral hypoglycemic agent ≥ 3, and duration of type 2 diabetes ≥ 5 years

The P values listed in the tables represent the interaction between the subgroups.

Table S4. Subgroup analysis of the risk for non-osteoporotic fractures according to the presence of psoriasis and rheumatoid arthritis

Subgroup	PsO	No. of participants	No. of events	Total no. of person-years of follow up	IR (per 1,000 person-years)	Model 5 HR (95% CI)	*P for interaction*
Age							0.322
<65	No	1482567	73269	9975959.89	7.35	1 (Ref.)	
	Yes	13176	752	87206.94	8.62	1.14 (1.06, 1.22)	
≥65	No	618640	32327	3764511.27	8.59	1 (Ref.)	
	Yes	6517	362	38842.46	9.32	1.07 (0.96, 1.18)	
Sex							0.147
Male	No	1267810	65304	8363908.56	7.81	1 (Ref.)	
	Yes	13371	797	85958.98	9.27	1.15 (1.07, 1.23)	
Female	No	833397	40292	5376562.60	7.49	1 (Ref.)	
	Yes	6322	317	40090.42	7.91	1.04 (0.93, 1.16)	
Smoking							0.305
Non-smoker	No	1176674	57660	7658722.13	7.53	1 (Ref.)	
	Yes	9494	522	60302.50	8.66	1.12 (1.03, 1.22)	
Former smoker	No	412319	20502	2728610.04	7.51	1 (Ref.)	
	Yes	4814	250	31195.91	8.01	1.03 (0.91, 1.17)	
Current	No	512214	27434	3353139.00	8.18	1 (Ref.)	
	Yes	5385	342	34550.98	9.89	1.17 (1.06, 1.31)	
Alcohol consumption							0.584
Non	No	1217340	59615	7857287.64	7.59	1 (Ref.)	
	Yes	11572	619	72682.36	8.52	1.09 (1.01, 1.18)	
Mild	No	680107	33835	4539300.77	7.45	1 (Ref.)	
	Yes	6266	362	41168.31	8.79	1.14 (1.03, 1.26)	
Heavy	No	203760	12146	1343882.76	9.04	1 (Ref.)	
	Yes	1855	133	12198.72	10.90	1.19 (1.00,1.41)	
Regular physical activity							0.557
No	No	1647130	82422	10712227.02	7.69	1 (Ref.)	
	Yes	15326	868	97319.03	8.92	1.13 (1.05, 1.20)	
Yes	No	454077	23174	3028244.15	7.65	1 (Ref.)	
	Yes	4367	246	28730.36	8.56	1.08 (0.95, 1.22)	
Duration of type 2 diabetes							0.333
<5 years	No	1431613	66892	9417602.75	7.10	1 (Ref.)	
	Yes	12965	697	83934.45	8.30	1.14 (1.06, 1.23)	
≥5 years	No	669594	38704	4322868.41	8.95	1 (Ref.)	
	Yes	6728	417	42114.94	9.90	1.07 (0.98, 1.18)	
	RA						
Age							<.001
<65	No	1456938	72037	9809317.33	7.34	1 (Ref.)	
	Yes	38805	1984	253849.50	7.82	1.02 (0.98, 1.07)	
≥65	No	598266	31636	3645656.51	8.68	1 (Ref.)	
	Yes	26891	1053	157697.22	6.68	0.76 (0.72, 0.81)	
Sex							<.001
Male	No	1257075	64708	8298754.20	7.80	1 (Ref.)	
	Yes	24106	1393	151113.35	9.22	1.11 (1.05, 1.17)	
Female	No	798129	38965	5156219.64	7.56	1 (Ref.)	
	Yes	41590	1644	260433.38	6.31	0.80 (0.76, 0.84)	
Smoking							<.001
Non-smoker	No	1138456	56189	7419864.91	7.57	1 (Ref.)	
	Yes	47712	1993	299159.72	6.66	0.84 (0.80, 0.88)	
Former smoker	No	408065	20240	2702638.90	7.49	1 (Ref.)	
	Yes	9068	512	57167.05	8.96	1.12 (1.03, 1.22)	
Current	No	508683	27244	3332470.03	8.18	1 (Ref.)	
	Yes	8916	532	55219.96	9.63	1.10 (1.01, 1.20)	
Alcohol consumption							<.001
Non	No	1179667	58135	7624205.14	7.63	1 (Ref.)	
	Yes	49245	2099	305764.86	6.86	0.86 (0.82, 0.89)	
Mild	No	673203	33471	4495533.04	7.45	1 (Ref.)	
	Yes	13170	726	84936.04	8.55	1.09 (1.01, 1.17)	
Heavy	No	202334	12067	1335235.65	9.04	1 (Ref.)	
	Yes	3281	212	20845.83	10.17	1.07 (0.94, 1.23)	
Regular physical activity							0.363
No	No	1609762	80842	10481565.51	7.71	1 (Ref.)	

	Yes	52694	2448	327980.55	7.46	0.92 (0.89, 0.96)	
Yes	No	445442	22831	2973408.33	7.68	1 (Ref.)	
	Yes	13002	589	83566.18	7.05	0.89 (0.82, 0.96)	
Duration of type 2 diabetes							0.968
<5 years	No	1403144	65823	9240373.83	7.12	1 (Ref.)	
	Yes	41434	1766	261163.38	6.76	0.92 (0.87, 0.96)	
≥5 years	No	652060	37850	4214600.01	8.98	1 (Ref.)	
	Yes	24262	1271	150383.35	8.45	0.92 (0.87, 0.97)	

PsO, psoriasis; RA, rheumatoid arthritis; IR, incidence rate; HR, hazard ratio; CI, confidence interval

Model 5 adjusted for age, sex, smoking, alcohol consumption, regular physical activity, hypertension, dyslipidemia, BMI, depression, use of insulin, number of oral hypoglycemic agent ≥ 3, and duration of type 2 diabetes ≥ 5 years

The P values listed in the tables represent the interaction between the subgroups.

Table S5. Subgroup analysis of the risk for vertebral fractures according to the presence of psoriasis and rheumatoid arthritis

Subgroup	PsO	No. of participants	No. of events	Total no. of person-years of follow up	IR (per 1,000 person-years)	Model 5 HR (95% CI)	*P for interaction*
Age							0.052
<65	No	1482567	25182	10297443.11	2.45	1(Ref.)	
	Yes	13176	313	90369.17	3.46	1.41 (1.26, 1.57)	
≥65	No	618640	39744	3988780.89	9.96	1(Ref.)	
	Yes	6517	439	41124.99	10.67	1.22 (1.11, 1.34)	
Sex							0.033
Male	No	1267810	21176	8598863.24	2.46	1(Ref.)	
	Yes	13371	356	88868.21	4.01	1.40 (1.26, 1.56)	
Female	No	833397	43750	5687360.76	7.69	1(Ref.)	
	Yes	6322	396	42625.95	9.29	1.20 (1.09, 1.32)	
Smoking							0.354
Non-smoker	No	1176674	49456	8027709.67	6.16	1(Ref.)	
	Yes	9494	482	63567.98	7.58	1.25 (1.14, 1.36)	
Former smoker	No	412319	7089	2805292.99	2.53	1(Ref.)	
	Yes	4814	133	32129.12	4.14	1.43 (1.20, 1.69)	
Current	No	512214	8381	3453221.35	2.43	1(Ref.)	
	Yes	5385	137	35797.06	3.83	1.33 (1.13, 1.58)	
Alcohol consumption							0.555
Non	No	1217340	50354	8226268.23	6.12	1(Ref.)	
	Yes	11572	566	76277.04	7.42	1.28 (1.18, 1.39)	
Mild	No	680107	11352	4672037.16	2.43	1(Ref.)	
	Yes	6266	150	42562.73	3.52	1.36 (1.16, 1.60)	
Heavy	No	203760	3220	1387918.61	2.32	1(Ref.)	
	Yes	1855	36	12654.39	2.84	1.12 (0.80, 1.55)	
Regular physical activity							0.174
No	No	1647130	53281	11142424.37	4.78	1(Ref.)	
	Yes	15326	625	101688.23	6.15	1.32 (1.22, 1.43)	
Yes	No	454077	11645	3143799.63	3.70	1(Ref.)	
	Yes	4367	127	29805.94	4.26	1.15 (0.97, 1.37)	
Duration of type 2 diabetes							0.346
<5 years	No	1431613	39076	9739768.43	4.01	1(Ref.)	
	Yes	12965	427	87003.79	4.91	1.25 (1.14, 1.38)	
≥5 years	No	669594	25850	4546455.57	5.69	1(Ref.)	
	Yes	6728	325	44490.37	7.30	1.34 (1.20, 1.50)	
	RA						
Age							<.001
<65	No	1456938	24190	10121774.60	2.39	1(Ref.)	
	Yes	38805	1305	266037.68	4.90	1.42 (1.35, 1.50)	
≥65	No	598266	37763	3860277.66	9.78	1(Ref.)	
	Yes	26891	2420	169628.22	14.27	1.24 (1.19, 1.29)	
Sex							0.003
Male	No	1257075	20799	8530840.35	2.44	1(Ref.)	
	Yes	24106	733	156891.10	4.67	1.47 (1.37, 1.58)	
Female	No	798129	41154	5451211.91	7.55	1(Ref.)	
	Yes	41590	2992	278774.80	10.73	1.26 (1.22, 1.31)	
Smoking							0.015
Non-smoker	No	1138456	46797	7772667.55	6.02	1(Ref.)	
	Yes	47712	3141	318610.10	9.86	1.27 (1.23, 1.32)	
Former smoker	No	408065	6943	2777946.08	2.50	1(Ref.)	
	Yes	9068	279	59476.03	4.69	1.40 (1.24, 1.58)	
Current	No	508683	8213	3431438.64	2.39	1(Ref.)	
	Yes	8916	305	57579.77	5.30	1.49 (1.33, 1.68)	
Alcohol consumption							0.497
Non	No	1179667	47696	7977180.60	5.98	1(Ref.)	
	Yes	49245	3224	325364.67	9.91	1.29 (1.24, 1.34)	
Mild	No	673203	11086	4626045.55	2.40	1(Ref.)	
	Yes	13170	416	88554.34	4.70	1.37 (1.24, 1.51)	
Heavy	No	202334	3171	1378826.11	2.30	1(Ref.)	
	Yes	3281	85	21746.89	3.91	1.33 (1.07, 1.64)	
Regular physical activity							0.373
No	No	1609762	50755	10896547.65	4.66	1(Ref.)	

	Yes	52694	3151	347564.95	9.07	1.31 (1.26, 1.36)	
Yes	No	445442	11198	3085504.61	3.63	1(Ref.)	
	Yes	13002	574	88100.95	6.52	1.26(1.15, 1.37)	
Duration of type 2 diabetes							0.004
<5 years	No	1403144	37264	9552326.10	3.90	1(Ref.)	
	Yes	41434	2239	274446.13	8.16	1.35 (1.30, 1.41)	
≥5 years	No	652060	24689	4429726.16	5.57	1(Ref.)	
	Yes	24262	1486	161219.78	9.21	1.23 (1.16, 1.29)	

PsO, psoriasis; RA, rheumatoid arthritis; IR, incidence rate; HR, hazard ratio; CI, confidence interval
Model 5 adjusted for age, sex, smoking, alcohol consumption, regular physical activity, hypertension, dyslipidemia, BMI, depression, use of insulin, number of oral hypoglycemic agent ≥ 3, and duration of type 2 diabetes ≥ 5 years
The P values listed in the tables represent the interaction between the subgroups.

Table S6. Subgroup analysis of the risk for hip fractures according to the presence of psoriasis and rheumatoid arthritis

Subgroup	PsO	No. of participants	No. of events	Total no. of person-years of follow up	IR (per 1,000 person-years)	Model 5 HR (95% CI)	*P for interaction*
Age							0.063
<65	No	1482567	5774	10363328.59	0.56	1 (Ref.)	
	Yes	13176	77	91161.23	0.84	1.40 (1.12, 1.76)	
≥65	No	618640	20027	4071252.80	4.92	1 (Ref.)	
	Yes	6517	211	42075.06	5.01	1.09 (0.96, 1.25)	
Sex							0.528
Male	No	1267810	9327	8642642.78	1.08	1 (Ref.)	
	Yes	13371	137	89642.96	1.53	1.12 (0.94, 1.32)	
Female	No	833397	16474	5791938.61	2.84	1 (Ref.)	
	Yes	6322	151	43593.33	3.46	1.21 (1.03, 1.41)	
Smoking							0.901
Non-smoker	No	1176674	19007	8144092.39	2.33	1 (Ref.)	
	Yes	9494	179	64727.93	2.77	1.16 (1.00, 1.34)	
Former smoker	No	412319	2965	2820423.48	1.05	1 (Ref.)	
	Yes	4814	51	32409.54	1.57	1.23 (0.93, 1.62)	
Current	No	512214	3829	3470065.52	1.10	1 (Ref.)	
	Yes	5385	58	36098.82	1.61	1.13 (0.87, 1.46)	
Alcohol consumption							0.746
Non	No	1217340	20792	8340863.73	2.49	1 (Ref.)	
	Yes	11572	229	77563.23	2.95	1.17 (1.03, 1.33)	
Mild	No	680107	3837	4698797.17	0.82	1 (Ref.)	
	Yes	6266	43	42936.72	1.00	1.05 (0.78, 1.42)	
Heavy	No	203760	1172	1394920.50	0.84	1 (Ref.)	
	Yes	1855	16	12736.34	1.26	1.28 (0.78, 2.10)	
Regular physical activity							0.812
No	No	1647130	21741	11263348.36	1.93	1 (Ref.)	
	Yes	15326	240	103125.24	2.33	1.17 (1.03, 1.33)	
Yes	No	454077	4060	3171233.04	1.28	1 (Ref.)	
	Yes	4367	48	30111.05	1.59	1.13 (0.85, 1.50)	
Duration of type 2 diabetes							0.139
<5 years	No	1431613	12302	9837242.03	1.25	1 (Ref.)	
	Yes	12965	119	88101.24	1.35	1.05 (0.88, 1.26)	
≥5 years	No	669594	13499	4597339.36	2.94	1 (Ref.)	
	Yes	6728	169	45135.05	3.74	1.26 (1.08, 1.46)	
	RA						
Age							0.079
<65	No	1456938	5568	10184936.61	0.55	1 (Ref.)	
	Yes	38805	283	269553.21	1.05	1.31 (1.16, 1.47)	
≥65	No	598266	19116	3938575.12	4.85	1 (Ref.)	
	Yes	26891	1122	174752.74	6.42	1.16 (1.09, 1.23)	
Sex							0.955
Male	No	1257075	9166	8573883.80	1.07	1 (Ref.)	
	Yes	24106	298	158401.95	1.88	1.19 (1.06, 1.33)	
Female	No	798129	15518	5549627.94	2.80	1(Ref.)	
	Yes	41590	1107	285904	3.87	1.19 (1.12, 1.27)	
Smoking							0.480
Non-smoker	No	1138456	18021	7882793.42	2.29	1 (Ref.)	
	Yes	47712	1165	326026.89	3.57	1.19 (1.12, 1.26)	
Former smoker	No	408065	2895	2792856.62	1.04	1 (Ref.)	
	Yes	9068	121	59976.41	2.02	1.31 (1.09, 1.57)	
Current	No	508683	3768	3447861.70	1.09	1(Ref.)	
	Yes	8916	119	58302.64	2.04	1.12 (0.93, 1.35)	
Alcohol consumption							0.607
Non	No	1179667	19763	8085642.44	2.44	1 (Ref.)	
	Yes	49245	1258	332784.52	3.78	1.20 (1.13, 1.27)	
Mild	No	673203	3765	4652155.76	0.81	1 (Ref.)	
	Yes	13170	115	89578.12	1.28	1.09 (0.90, 1.31)	
Heavy	No	202334	1156	1385713.53	0.83	1 (Ref.)	
	Yes	3281	32	21943.31	1.46	1.24 (0.88, 1.77)	
Regular physical activity							0.280
No	No	1609762	20783	11011610.91	1.89	1 (Ref.)	

	Yes	52694	1198	354862.68	3.38	1.18 (1.11, 1.25)	
Yes	No	445442	3901	3111900.82	1.25	1 (Ref.)	
	Yes	13002	207	89443.26	2.31	1.28 (1.11, 1.47)	
Duration of type 2 diabetes							0.004
<5 years	No	1403144	11721	9645261.68	1.22	1 (Ref.)	
	Yes	41434	700	280081.59	2.50	1.29 (1.20, 1.40)	
≥5 years	No	652060	12963	4478250.05	2.89	1 (Ref.)	
	Yes	24262	705	164224.36	4.29	1.10 (1.02, 1.19)	

PsO, psoriasis; RA, rheumatoid arthritis; IR, incidence rate; HR, hazard ratio; CI, confidence interval

Model 5 adjusted for age, sex, smoking, alcohol consumption, regular physical activity, hypertension, dyslipidemia, BMI, depression, use of insulin, number of oral hypoglycemic agent ≥ 3, and duration of type 2 diabetes ≥ 5 years

The P values listed in the tables represent the interaction between the subgroups.
